# Synthesis of a Conformationally Fixed Bicyclomarin Derivative

**DOI:** 10.1002/cmdc.202500493

**Published:** 2025-09-03

**Authors:** Jennifer Greve, Alexander F. Kiefer, Uli Kazmaier

**Affiliations:** ^1^ Organic Chemistry Saarland University Campus C4.2 66123 Saarbrücken Germany; ^2^ Helmholtz Institute for Pharmaceutical Research Saarland (HIPS) Campus E8.1 66123 Saarbrücken Germany

**Keywords:** clpC1, cyclomarin, cyclopeptides, macrolactamizations, tuberculosis

## Abstract

Based on an X‐ray structure of cyclomarin bound to ClpC1, a new conformationally fixed, bicyclic cyclomarin derivative is synthesized in an effort to enhance antituberculosis activity. The synthesis of the linear heptapeptide and the two macrolactamizations proceed smoothly. Only the very last synthetic step, the cleavage of a benzyl ether, provides a low yield. Despite the successful synthesis, the resulting bicyclic compound shows reduced activity compared to cyclomarin.

## Graphical abstract

The synthesis of a new bicyclic derivative of cyclomarin is described. Cyclomarin is a marine cyclopeptide with excellent antituberculosis activity. The synthesis of the linear heptapeptide and the two macrolactamizations proceed smoothly.

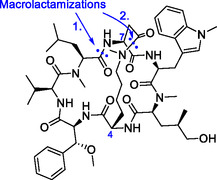

## 1 Introduction

Marine organisms are prolific sources of often biologically highly active natural products.^[^ ^1^ ^]^ Many of these compounds show remarkable anticancer or antimicrobial activity, making them ideal candidates for drug development, including treatments for infectious diseases, like tuberculosis (TB).^[^ ^2^ ^]^ In 2023, ≈10.8 million people developed TB, resulting in 1.25 million deaths.^[^ ^3^ ^]^ The biggest problem in the fight against this disease is the continuous development of resistant strains of *Mycobacterium tuberculosis* (*M. tub.*), the pathogen. In 2022, 450,000 people were infected by strains resistant to the most effective first‐line drug rifampicin, and 80% of them suffered from multidrug‐resistant tuberculosis (MDR‐TB).^[^ ^3b^ ^]^ Therefore, there is an urgent need to develop new drugs that are also effective against the largely drug‐resistant tuberculosis strains (XDR‐TB) due to new mechanisms of action.^[^ ^4^ ^]^ In this context, natural products are of particular importance, as more than 60% of the anti‐TB drugs currently developed are natural products or derived from them.^[^ ^5^ ^]^

**Figure 1:**
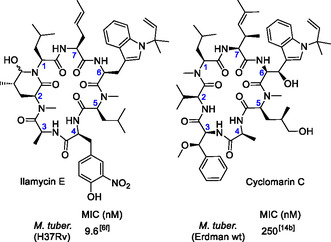
Marine cyclopeptides with antituberculosis activity.

From 1962 on, several research groups isolated a series of cyclic peptides, namely the structurally closely related ilamycins (Ila) (**Figure** **1**) and rufomycins (Ruf), from marine *Streptomyces* sp., exhibiting notable activity against Mycobacteria.^[^ ^6^ ^]^ Interestingly, these compounds exhibit little to no activity against most other gram‐positive and gram‐negative bacteria, fungi, or yeasts.^[^ ^6f^ ^]^

In 1999, the Fenical and Clardy groups reported the isolation of three cyclomarins (Cym) A−C from extracts of a marine *Streptomyces* sp. (CNB‐982).^[^ ^7^ ^]^ These compounds are structurally related to ilamycins/rufomycins.^[^ ^8^ ^]^ They contain very similar amino acid building blocks, but interestingly, in a different order. As in the ilamycins, an unusual *N*‐prenylated tryptophan is found here (CymC) (Figure 1), which can also be epoxidized (CymA).

However, unlike in Ilas and Rufs, this tryptophan unit is further modified by *β*‐oxidation in Cyms. A *γ*,*δ*‐unsaturated amino acid is also incorporated into the *N*‐terminus of tryptophan, and one of the leucines is oxidized at the *δ* position, but at a different site, as in ilamycins.

Cyclomarins are also active against drug‐resistant *Mtb* in the high nanomolar range.^[^ ^9^ ^]^ They bind to the *N*‐terminal domain of the hexameric AAA^+^ ATPase ClpC1, which acts together with the associated ClpP1P2 protease complex in protein degradation. The *N*‐terminal domain ClpC1 is responsible for the recognition, unfolding, and translocation of protein substrates into the ClpP1P2 protease for the degradation of superfluous and unwanted proteins.^[^ ^10^ ^]^ Cyclomarins appear to interfere with the arginine‐phosphate‐induced dynamics in the *N*‐terminal domain of ClpC1 and thereby disrupting protein degradation.^[^ ^11^ ^]^

Their excellent biological activities drove the development of syntheses of these interesting cyclic peptides. So far, only one synthesis for the ilamycins E and F has been reported by Guo and Ye et al. in 2018;^[^ ^12^ ^]^ the first synthesis of CymC was described by Yao and colleagues in 2004.^[^ ^13^ ^]^ Further syntheses of CymA, CymC, and CymD were reported by our group,^[^ ^14^ ^]^ which also synthesized a range of derivatives of CymC^[^ ^15^ ^]^ and IlaE.^[^ ^16^ ^]^ Building on the promising antitubercular activities and the unique mode of action of the natural products and their derivatives, a series of BacPROTACs has been developed that initiate targeted protein degradation.^[^ ^17^ ^]^

## 2 Results and Discussion

**Figure 2:**
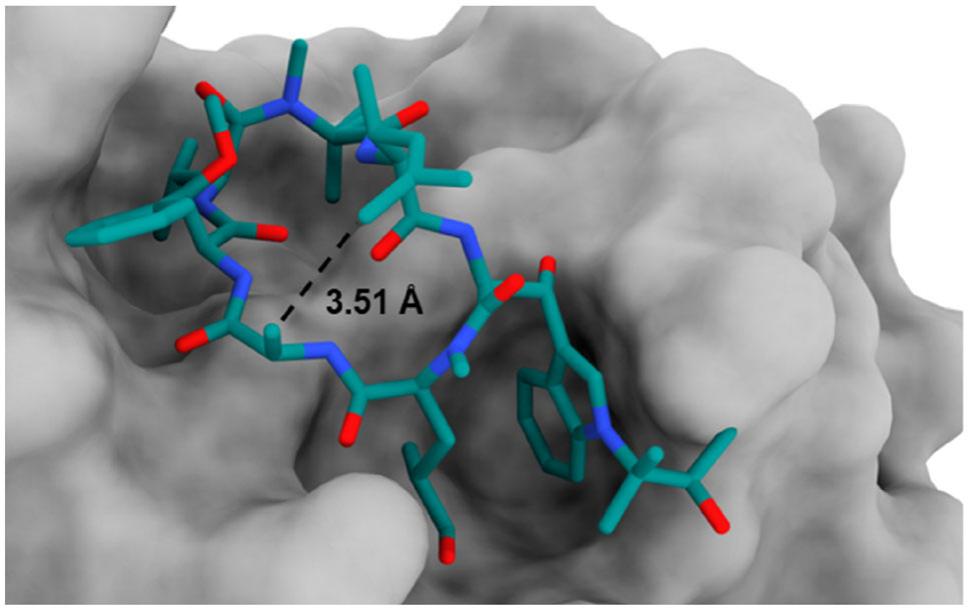
Cyclomarin A (teal) bound to *N*‐terminal domain of *M. tuberculosis* ClpC1 (gray) (PDB: 3WDC). Image was generated with UCSF ChimeraX 1.8 software.

**Figure 3:**
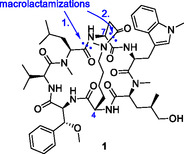
Planned bicyclic cyclomarin derivative **1**.

Herein, the synthesis of a conformationally fixed bicyclic cyclomarin derivative **1** (Figure 3) is described, from which we hoped to achieve an even higher binding affinity to ClpC1. Based on a crystal structure of cyclomarin A bound to ClpC1,^[^ ^18^ ^]^ we decided to introduce a bridge between the alanine (fourth amino acid) and the unsaturated amino acid (seventh amino acid), since those residues are in close proximity of 3.51 Å (**Figure** **2**).

Since this linkage should be installed at late stage, we wanted to circumvent the utilization of reactions that involve transition metals such as ruthenium or molybdenum used in ring‐closure metathesis or copper‐catalyzed azide–alkyne cycloaddition to avoid any interference in the downstream bioactivity testing of this compound. Therefore, we chose the most simple connection strategy, a macrolactamization, enabled by the replacement of alanine to lysine and *γ*,*δ*‐unsaturated amino acid to aspartic acid. Accordingly, the lysine building block should be *N*‐methylated in the side chain to possibly improve cell permeability (**Figure** **3**).

The synthesis of the bicyclic cyclomarin derivative was planned in analogy to the synthesis of other cyclomarins,^[^ ^14b^ ^]^ following a linear linking strategy from the *C*‐ to *the N‐*terminus with subsequent cyclization between amino acids 1 and 7, followed by a second macrolactamization between the lysine at position 4 and the aspartate at position 7. Since the derivative should be derived from deoxycyclomarin C, the *β*‐hydroxy group in tryptophan is also missing here. In addition, because the *N*‐substituent on the indole moiety had no significant influence on the anti‐TB activity in our previous investigations,^[^ ^15c^ ^]^ a methylated indole was used instead of the reversed *tert*‐prenylated one.

Since the deprotection of the primary alcohol in the hydroxyleucine unit should take place after the second macrolactamization step, a benzyl protection group was chosen for this purpose. The amino acid building block required could be obtained by simple protecting group interconversion of the known *N‐*methylated tert‐Butyldimethylsilyl (TBS)‐protected hydroxyleucine derivative.^[^ ^15b^ ^]^ The other amino acid building blocks were also obtained according to literature.

**Scheme 1:**
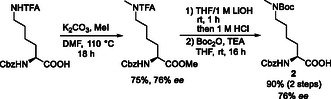
Synthesis of lysine building block **2**.

The modified lysine required for the additional bridging could be obtained from benzyloxycarbonyl (Cbz)‐Lys(trifluoroacetyl (TFA))‐OH only with a moderate enantiomeric excess of 76% (**Scheme** **1**). The reason for this was the racemization‐prone conditions (110 °C) of the methylation reaction carried out as the first step.^[^ ^19^ ^]^ Under these conditions, 15% *N‐*methylation of the Cbz‐amide was also observed, which, however, could be separated without any problems. Unfortunately, a more selective reaction by reducing the reaction temperature was not successful, since at lower temperatures, almost exclusively, the methylation of the carboxylic acid took place, while the amide functions remained untouched. Simultaneous saponification of the methyl ester and the TFA‐amide yielded the protected amino acid **2** after Boc protection of the *ε*‐amino function.

**Scheme 2:**
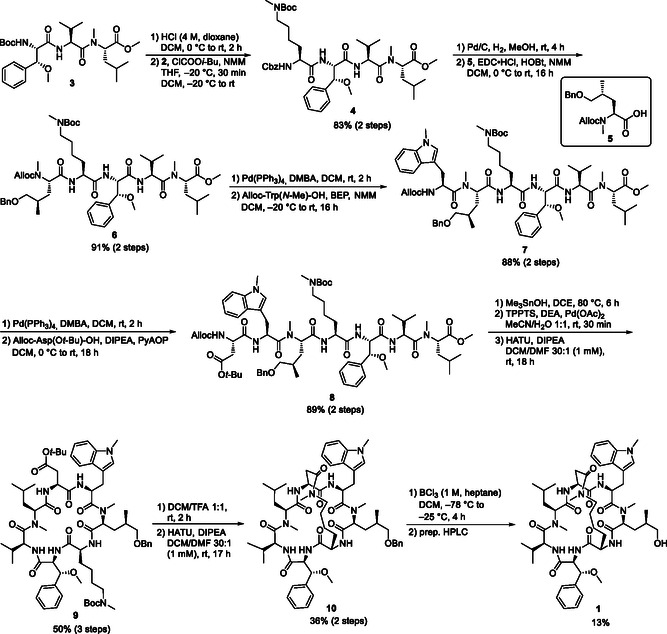
Synthesis of bicyclic cyclomarin derivative **1**.

In the next step, **2** was coupled with the known tripeptide **3** (**Scheme** **2**).^[^ ^14a^ ^]^ After incorporation of the modified lysine into the linear peptide, the undesired epimer could be easily separated by column chromatography, and the tetrapeptide **4** could be obtained in diastereomerically pure form. After hydrogenolytic cleavage of the Cbz protection group, the free amine was linked to the benzyl‐protected hydroxyleucine building block **5** to form pentapeptide **6** in very good yield. Subsequent removal of the Alloc protecting group, using 1,3 dimethylbarbituric acid (DMBA) as allyl scavenger, and coupling of the secondary amine obtained to the tryptophan moiety using BEP (2‐bromo‐1‐ethyl pyridinium tetrafluoroborate) yielded the desired hexapeptide **7**. By renewed palladium‐catalyzed cleavage of the amine protection group and reaction with the aspartate building block with PyAOP (7‐azabenzotriazole‐1‐yloxy)tripyrrolidinophosphonium hexafluorophosphate) as a coupling reagent, the linear heptapeptide **8** was obtained in a good overall yield of about 60% (over eight steps) starting from tripeptide **3**.

Subsequently, the linear heptapeptide methyl ester **8** was saponified with Me_3_SnOH at 80 °C.^[^ ^20^ ^]^ Despite the mild reaction conditions, however, it was not possible to completely suppress the cleavage of the *tert*‐butyl ester of the aspartate. The undesirable side product had to be separated by column chromatography, as it impaired the formation of the desired macrocycle. After deprotecting of the *N‐*terminus, the linear peptide was cyclized to the monocyclic cyclomarin derivative **9** under high‐dilution conditions.

Trifluoroacetic acid in dichloromethane (DCM) was used to cleave both the Boc protecting group of the lysine and the *tert*.‐butyl ester of the aspartate. Subsequent second macrolactamization, also under high‐dilution conditions, yielded the desired bicyclus **10** with an acceptable yield of 36% over two steps.

However, the final benzyl deprotection turned out to be far from trivial. In the hydrogenolytic cleavage tested at the beginning, no turnover was observed, neither with Pd/C nor with the more reactive Pearlman catalyst (Pd(OH)_2_). An increase in pressure to 100 bar, an increase in temperature to 100 °C and even the use of stoichiometric quantities of catalyst did not lead to any successful deprotection. Therefore, a Lewis acid‐based approach was chosen next. The aim was to selectively cleave the primary benzyl ether in the presence of a secondary benzylic methyl ether (amino acid 3). Panek and Xu succeeded in a similar situation during their total synthesis of (+)‐macbecin I using boron trichloride.^[^ ^21^ ^]^ The conversion to primary alcohol was carried out in DCM with a 1 M boron trichloride solution at –78 °C. Since the first decomposition reactions could already be observed at a conversion of about 80% (LC/MS), the reaction was stopped at this point. However, the desired bicyclic cyclomarin derivative could only be isolated in 13% yield after purification via flash chromatography.

The quantities obtained were sufficient to carry out initial biological studies, as we wanted to evaluate whether the bicyclic derivative **1** shows comparable or even better activities than other monocyclic cyclomarin derivatives, which often have Minimum inhibitor concentration (MIC) values <1 μM (*Mtb* Erdman).^[^ ^15c^ ^]^ Antibacterial activity was assessed using *Mycobacterium smegmatis* mc^2^155, with rifampicin as a positive control, while cytotoxicity was evaluated against HepG2 cells using doxorubicin as a reference compound (**Table** **1**). Fortunately, compound **1** was noncytotoxic toward HepG2 cells; however, its activity against *M. smegmatis* was weak, leading us to discontinue further synthetic optimization.

**Table 1:** Biological evaluation of bicyclomarin 1.

Compound	MIC [μM]a) *M. smegmatis* mc^2^ 155	CC_50_ [μM]b) HepG2
Rifampicinc)	19.4d)	NDe)
Doxorubicinc)	NDe)	0.29 ± 0.1f)
Bicyclomarin (**1**)	64.9d)	>3f)
Cyclomarin A^[^ ^9b^ ^]^	0.6	ND

a) Complete growth inhibition of the organism (detected by naked eye);

b) concentration at which 50% of cells died;

c) positive control;

d) determined once;

e) ND: not determined;

f) determined twice.

## 3 Conclusion

In conclusion, we synthesized a new bicyclic cyclomarin derivative and investigated its biological activity. The aim was to synthesize a conformationally fixed derivative based on an X‐ray structure of cyclomarin bound to ClpC1, the natural target of this cyclopeptide, which binds even better to ClpC1 than cyclomarin itself. The synthesis of the linear heptapeptide was carried out in consistently very good yields. The two critical macrolactamizations also provided good results. Only the final step, the cleavage of a benzyl ether from this highly functionalized molecule, resulted in an only modest yield of the desired bicyclic cyclomarin derivative. Initial biological evaluations revealed that the antimycobacterial activity fell short of our expectations.

## Conflict of Interest

The authors declare no conflict of interest.

## Supporting information

Supplementary Material

## Data Availability

The data that support the findings of this study are available in the supplementary material of this article.
