# Supplementary material for: Synthesis of a Conformationally Fixed Bicyclomarin Derivative
Source: ChemMedChem. 2025 Sep 3;20(21):e202500493. doi: 10.1002/cmdc.202500493 (PMC12597213; doi:10.1002/cmdc.202500493)
Supplement: Supplementary file 1 — Supplementary Material [file CMDC-20-e202500493-s001.pdf]

Supporting Information

Abbreviation list	S1
General Information	S2
Synthesis of the Compounds	S3
Copies of the NMR spectra	S14
References	S22

Abbreviation list

Ac	acetyl
Alloc	allyloxycarbonyl
Asp	aspartic acid
BEP	2-bromo-1-ethyl-pyridinium-tetrafluoroborate
Boc	*tert*-butyloxycarbonyl
Cbz	benzyloxycarbonyl
CI	chemical ionization
DCE	1,2-dichloroethane
DCM	dichloromethane
DIPEA	*N*,*N*-diisopropylethylamine
DMF	*N*,*N*-dimethylformamide
DMSO	dimethyl sulfoxide
EDC	1-ethyl-3-(3-dimethyl-aminopropyl)carbodiimide
ESI	electrospray ionization
Et	ethyl
HATU	1-[bis(dimethylamino)methylene]-1*H*-1,2,3-triazolo[4,5-b]pyridinium 3-oxide hexafluorophosphate
HOBt	*N*-hydroxybenzotriazole
HPLC	high-performance liquid chromatography
HRMS	high-resolution mass spectrometry
IBCF	isobutyl chloroformate
LC/MS	liquid chromatography–mass spectrometry
Lys	lysine
NMM	*N*-methylmorpholine
PyAOP	(7-aza-benzotriazol-1-yloxy)tripyrrolidinophosphonium hexafluorophosphate
sat.	saturated
TFA	trifluoroacetic acid
THF	tetrahydrofuran
TPPTS	3,3′,3″-phosphanetriyltris(benzenesulfonic acid) trisodium salt
t_R	retention time
Trp	tryptophan

General Information

All air- and moisture-sensitive reactions were carried out in oven-dried reaction flasks (80 °C) under nitrogen atmosphere. Ethyl acetate was distilled before use. Anhydrous THF was prepared by distillation over sodium/benzophenone. All other anhydrous solvents were purchased from *Acros Organics*.

Reaction control was done by LC/MS analysis on a *Shimadzu* Prominence-i LC-2030C 3D Plus (*Phenomenex* Onyx® C18, 50 x 4.6 mm) coupled with *Shimadzu* LCMS-2020 (ESI ionization). All runs were performed at a flow rate of 4 mL/min and 0.1% $HCOOH_{aq}$/MeCN (90:10 to 1:99 (1.5 min), 1:99 (1 min), 90:10 (0.7 min)) as mobile phase with a column temperature of 40 °C.

For column chromatographic purification a *Büchi* Reveleris® Prep Chromatography System with *Büchi* FlashPure Select C18 cartridges were used. Preparative HPLC was done with a *Büchi* Reveleris® Prep chromatography system with a *Phenomenex* Luna® (C18, 250 x 21.1 mm, 5 µm).

NMR spectra were recorded with Bruker Avance II 400 [400 MHz (^{1}H), 100 MHz (^{13}C), 298 K], Bruker Avance I 500 or Bruker Avance Neo 500 [500 MHz (^{1}H), 125 MHz (^{13}C), 298 K]. Spectra were calibrated using the solvent signals $CDCl_3$ (^{1}H 7.26 ppm, ^{13}C 77.00 ppm), DMSO-d_6 (^{1}H 2.50 ppm, ^{13}C 39.51 ppm). The software ACD/NMR Processor Academic Edition (version 12.01) was used to analyze the spectra. Multiplicities in the ^{1}H-NMR spectra are reported as singlet (s), doublet (d), triplet (t), quartet (q) and multiplet (m). ^{13}C measurements were broadband decoupled and the theoretical multiplicity of the carbon is given as s (quaternary C atom), d (tertiary C atom), t (secondary C atom) and q (primary C atom). Peaks were assigned using H,H-COSY, HSQCED and HMBC spectra.

Optical rotation values were measured using either a *Jasco* P-2000 polarimeter or a *Krüss* P8000-T polarimeter with thermostated (20 °C ± 1 °C) 1 dm cuvette at the sodium D line (589 nm). Concentration c is given in g/100 mL and $[\alpha]_D^{20}$ values are given in 10^{-1} deg cm^2 g^{-1}.

Mass spectra (HRMS) were recorded using a *Bruker* Daltonics maXis 4G hr-ToF spectrometer (ESI) or a *Finnigan* MAT 95 sector field spectrometer (CI).

The protected amino acids (Cbz-Lys(Boc-*N*-Me)-OH (**2**),[1] Alloc-Trp(*N*-Me)-OH[2] and Alloc-Asp(O*t*-Bu)-OH[3]) as well as tripeptide **3**[4] were synthesized according to the literature.

Synthesis of the Compounds

Methyl *N*-(((2*S*,3*R*)-2-((*S*)-2-(((benzyloxy)carbonyl)amino)-6-((*tert*-butoxycarbonyl)(methyl)amino)hexanamido)-3-methoxy-3-phenylpropanoyl)-L-valyl)-*N*-methyl-L-leucinate (4)

Under nitrogen atmosphere, a solution of tripeptide **3**[4] (1.98 g, 3.70 mmol, 1.0 eq.) in 37 mL anhydrous DCM was cooled to 0 °C and HCl in 1,4-dioxane (18.5 mL, 74.0 mmol, 4.0 M, 20 eq.) was added. The ice bath was removed and reaction mixture was stirred at room temperature for two hours. After removing the solvent under reduced pressure, the residue was dried in high vacuum and then dissolved in 20 mL anhydrous DCM.

To a solution of Cbz-Lys(Boc-*N*-Me)-OH (1.61 g, 4.07 mmol, 1.1 eq.) in 14 mL anhydrous THF, NMM (ρ = 0.92 g/mL, 1.0 mL, 9.25 mmol, 2.5 eq.) and IBCF (ρ = 1.05 g/mL, 530 µL, 4.07 mmol, 1.1 eq.) were added dropwise at –20 °C. After stirring for 20 min at this temperature, the amine component solution was added. The reaction mixture was slowly warmed to room temperature (15 h, LC/MS control), hydrolyzed with water and diluted with EtOAc. Subsequently, the phases were separated and the organic phase was successively washed with 1 M $KHSO_{4aq}$, sat. $NaHCO_3$ solution and brine, dried over Na_2SO_4 and concentrated under reduced pressure. After purification by column chromatography (C18 spherical, H_2O/MeCN 9:1 to MeCN), tetrapeptide **4** (2.49 g, 3.06 mmol, 83%) was isolated as a colorless foam.

LC/MS: t_R (4) = 1.56 min

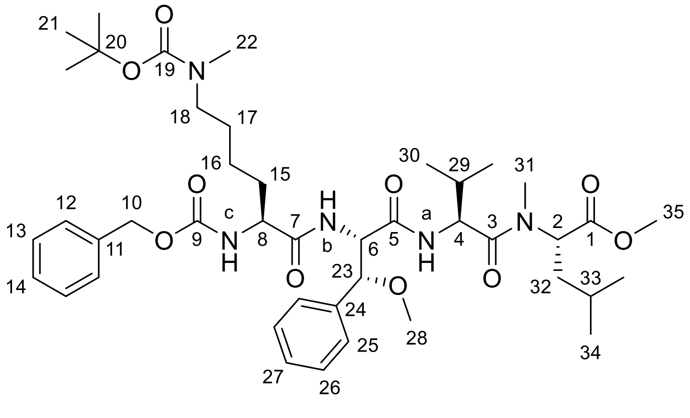

4

^{1}H-NMR (500 MHz, DMSO-d$_6$, 373 K): δ = 7.59 (d, $^3J_{NHa,4}$ = 8.7 Hz, 1 H, NH$_a$), 7.51 (d, $^3J_{NHb,6}$ = 6.9 Hz, 1 H, NH$_b$), 7.30 (m, 10 H, 12-H, 13-H, 14-H, 25-H, 26-H, 27-H), 6.99 (d, $^3J_{NHc,8}$ = 4.9 Hz, 1 H, NH$_c$), 5.09 (d, $^2J_{10,10'}$ = 12.7 Hz, 1 H, 10-H), 5.08 (m, 1 H, 2-H), 5.05 (d, $^2J_{10',10}$ = 12.7 Hz, 1 H, 10'-H), 4.73 (d, $^3J_{23,6}$ = 3.2 Hz, 1 H, 23-H), 4.67 (dd, $^3J_{4,NHa}$ = 8.7 Hz, $^3J_{4,29}$ = 7.3 Hz, 1 H, 4-H), 4.60 (dd, $^3J_{6,NHb}$ = 8.7 Hz, $^3J_{6,23}$ = 3.2 Hz, 1 H, 6-H), 4.05 (td, $^3J_{8,15}$ = 8.5 Hz, $^3J_{8,NHc}$ = 5.3 Hz, 1 H, 8-H), 3.64 (s, 3 H, 35-H), 3.19 (s, 3 H, 28-H), 3.11 (t, $^3J_{18,17}$ = 7.2 Hz, 2 H, 18-H), 2.95 (m, 3 H, 31-H), 2.75 (s, 3 H, 22-H), 2.07 (m, 1 H, 29-H), 1.69 (m, 2 H, 32-H), 1.49 (m, 5 H, 15-H, 17-H, 33-H), 1.40 (s, 9 H, 21-H), 1.19 (m, 2 H, 16-H), 0.93 (d, $^3J_{30,29}$ = 6.9 Hz, 3 H, 30-H), 0.91 (d, $^3J_{30',29}$ = $^3J_{34,33}$ = 6.7 Hz, 6 H, 30'-H, 34-H), 0.88 (d, $^3J_{34',33}$ = 6.0 Hz, 3 H, 34'-H).

^{13}C-NMR (125 MHz, DMSO-d$_6$, 373 K): δ = 171.1 (s, C-7), 171.1 (s, C-3), 170.9 (s, C-1), 168.1 (s, C-5), 155.2 (s, C-9), 154.4 (s, C-19), 137.6 (s, C-24), 136.6 (s, C-11), 127.7 (d, C-13), 127.3

(d, C-26), 127.1 (d, C-14), 127.0 (d, C-12), 126.9 (d, C-27), 126.5 (d, C-25), 81.7 (d, C-23), 77.7 (s, C-20), 65.1 (t, C-10), 56.9 (d, C-4), 56.4 (q, C-28), 54.6 (d, C-8), 54.2 (d, C-2), 53.3 (d, C-6), 51.1 (q, C-35), 47.5 (t, C-18), 36.4 (t, C-32), 33.2 (q, C-22), 31.1 (t, C-15), 30.8 (q, C-31), 30.0 (d, C-29), 27.7 (q, C-21), 26.5 (t, C-17), 23.9 (d, C-33), 22.2 (q, C-34), 22.1 (t, C-16), 21.0 (q, C-34‘), 18.5 (q, C-30), 17.1 (q, C-30‘).

optical rotation: $[\alpha]_D^{20}$ = –47.3 (c = 1.0, $CHCl_3$)

**HRMS (ESI):**	calculated	found
$C_{43}H_{66}N_5O_{10}$ $[M+H]^+$	812.4804	812.4812

(2*S*,4*R*)-2-(((Allyloxy)carbonyl)(methyl)amino)-5-(benzyloxy)-4-methylpentanoic acid (5)

The benzyl-protected δ-hydroxyleucine was obtained by protecting group exchange from the TBS-protected δ-hydroxyleucine.[5]

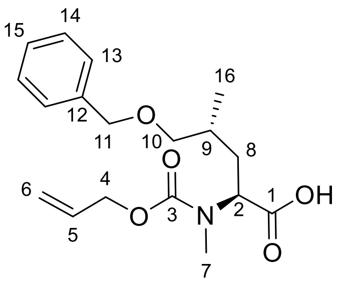

5

^{1}H-NMR (500 MHz, $CDCl_3$): δ = 7.31 (m, 5 H, 13-H, 14-H, 15-H), 5.94 (ddt, $^3J_{5,6}$ = 17.0 Hz, $^3J_{5,6'}$ = 10.7 Hz, $^3J_{5,4}$ = 5.4 Hz, 1 H, 5-H), 5.31 (d, $^3J_{6,5}$ = 17.3 Hz, 1 H, 6-H), 5.22 (d, $^3J_{6',5}$ = 10.7 Hz, 1 H, 6‘-H), 4.87 (dd, $^3J_{2,8}$ = 10.1 Hz, $^3J_{2,8'}$ = 5.4 Hz, 1 H, 2-H), 4.62 (d, $^3J_{4,5}$ = 4.4 Hz, 2 H, 4-H), 4.50 (s, 2 H, 11-H), 3.36 (m, 2 H, 10-H), 2.88 (s, 3 H, 7-H), 2.10 (ddd, $^2J_{8',8}$ = 13.9 Hz, $^3J_{8',9}$ = 8.2 Hz, $^3J_{8',2}$ = 5.4 Hz, 1 H, 8‘-H), 1.84 (m, 1 H, 9-H), 1.67 (ddd, $^2J_{8,8'}$ = 14.2 Hz, $^3J_{8,2}$ = 10.1 Hz, $^3J_{8,9}$ = 5.4 Hz, 1 H, 8-H), 1.02 (d, $^3J_{16,9}$ = 6.6 Hz, 3 H, 16-H).

^{13}C-NMR (100 MHz, $CDCl_3$): δ = 176.8 (s, C-1), 156.9 (s, C-3), 138.5 (s, C-12), 132.7 (d, C-5), 128.3 (d, C-14), 127.6 (d, C-13), 127.5 (d, C-15), 117.4 (t, C-6), 74.3 (t, C-10), 73.0 (t, C-11), 66.5 (t, C-4), 57.0 (d, C-2), 32.5 (t, C-8), 30.8 (q, C-7), 30.5 (d, C-9), 17.9 (q, C-16).

Selected rotamer signals:

^{1}H-NMR (500 MHz, $CDCl_3$): δ = 5.85 (ddt, $^3J_{5,6}$ = 17.0 Hz, $^3J_{5,6'}$ = 10.7 Hz, $^3J_{5,4}$ = 5.4 Hz, 1 H, 5-H), 5.25 (m, 1 H, 6-H), 5.17 (d, $^3J_{6',5}$ = 10.7 Hz, 1 H, 6‘-H), 4.80 (dd, $^3J_{2,8}$ = 10.1 Hz, $^3J_{2,8'}$ = 5.1 Hz, 1 H, 2-H), 4.56 (dd, $^3J_{4,5}$ = $^4J_{4,6}$ = 5.2 Hz, 2 H, 4-H), 4.48 (s, 2 H, 11-H), 1.01 (d, $^3J_{16,9}$ = 5.4 Hz, 3 H, 16-H).

^{13}C-NMR (100 MHz, $CDCl_3$): δ = 177.0 (s, C-1), 156.1 (s, C-3), 138.4 (s, C-12), 132.6 (d, C-5), 128.4 (d, C-14), 127.5 (d, C-13), 127.5 (d, C-15), 117.5 (t, C-6), 66.4 (t, C-4), 56.8 (d, C-2), 33.2 (t, C-8), 31.0 (q, C-7), 30.3 (d, C-9), 18.0 (q, C-16).

**HRMS (CI):**	calculated	found
$C_{18}H_{26}NO_5$ $[M+H]^+$	336.1805	336.1851

Methyl *N*-(((2*S*,3*R*)-2-((*S*)-2-((2*S*,4*R*)-2-(((allyloxy)carbonyl)(methyl)amino)-5-(benzyl-oxy)-4-methylpentanamido)-6-((*tert*-butoxycarbonyl)(methyl)amino)hexanamido)-3-methoxy-3-phenylpropanoyl)-L-valyl)-*N*-methyl-L-leucinate (6)

To a solution of tetrapeptide **4** (50.0 mg, 62.0 µmol, 1.0 eq.) in 600 µL methanol, Pd/C (5.0 mg, 10% on activated charcoal) was added at room temperature and the mixture was stirred at 1 bar H_2 atmosphere for four hours. Subsequently, the reaction mixture was filtered through a pad of celite® and the solvent was removed under reduced pressure. The residue was dissolved in 600 µL anhydrous DCM and hydroxyleucine derivative **5** (22.7 mg, 68.0 µmol, 1.1 eq.) was added.

The reaction mixture was cooled to 0 °C before adding NMM (ρ = 0.92 g/mL, 16.2 µL, 148 µmol, 2.4 eq.), HOBt (10.4 mg, 68.0 µmol, 1.1 eq.) and EDC•HCl (13.0 mg, 68.0 µmol, 1.1 eq.) in succession. After slowly warmed to room temperature (16 h, LC/MS control), the reaction mixture was diluted with EtOAc. The organic phase was washed with 1 M $KHSO_{4aq}$, sat. $NaHCO_3$ solution and brine, dried over Na_2SO_4 and concentrated under reduced pressure. After purification by column chromatography (C18 spherical, H_2O/MeCN 9:1 to MeCN), pentapeptide **6** (56.0 mg, 56.0 µmol, 91%) was obtained as a colorless resin.

LC/MS: t_R (6) = 1.70 min

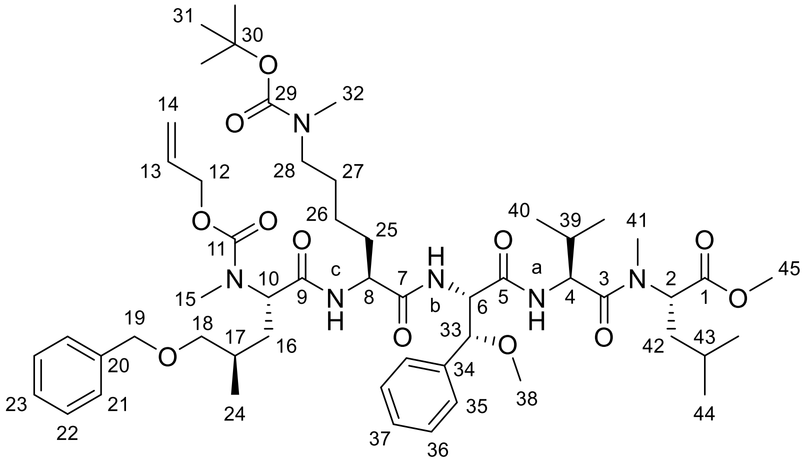

6

^{1}H-NMR (500 MHz, DMSO-d$_6$, 373 K): δ = 7.65 (d, $^3J_{NHb,6}$ = 7.6 Hz, 1 H, NH$_b$), 7.47 (m, 1 H, NH$_a$), 7.45 (d, $^3J_{NHc,8}$ = 8.2 Hz, 1 H, NH$_c$), 7.27 (m, 10 H, 21-H, 22-H, 23-H, 35-H, 36-H, 37-H), 5.93 (ddt, $^3J_{13,14}$ = 17.2 Hz, $^3J_{13,14'}$ = 10.5 Hz, $^3J_{13,12}$ = 5.3 Hz, 1 H, 13-H), 5.29 (dd, $^3J_{14,13}$ = 17.3 Hz, $^2J_{14,14'}$ = 1.6 Hz, 1 H, 14-H), 5.18 (dd, $^3J_{14',13}$ = 10.5 Hz, $^2J_{14',14}$ = 1.4 Hz, 1 H, 14'-H), 5.08 (m, 1 H, 2-H), 4.72 (d, $^3J_{33,6}$ = 3.2 Hz, 1 H, 33-H), 4.65 (dd, $^3J_{4,39}$ ≈ $^3J_{4,NHa}$ = 8.1 Hz , 1 H, 4-H), 4.62 (dd, $^3J_{10,16}$ = 9.3 Hz, $^3J_{10,16'}$ = 6.0 Hz, 1 H, 10-H), 4.57 (m, 1 H, 6-H), 4.56 (d, $^3J_{12,13}$ = 5.3 Hz, 2 H, 12-H), 4.46 (s, 2 H, 19-H), 4.31 (td, $^3J_{8,25}$ = 8.1 Hz, $^3J_{8,NHc}$ = 5.3 Hz, 1 H, 8-H), 3.63 (s, 3 H, 45-H), 3.42 (dd, $^2J_{18,18'}$ = 9.4 Hz, $^3J_{18,17}$ = 5.1 Hz, 1 H, 18-H), 3.28 (dd, $^2J_{18',18}$ = 9.4 Hz, $^3J_{18',17}$ = 6.8 Hz, 1 H, 18'-H), 3.18 (s, 3 H, 38-H), 3.10 (t, $^3J_{28,27}$ = 7.2 Hz, 2 H, 28-H), 2.94 (s, 3 H, 41-H), 2.80 (s, 3 H, 15-H), 2.75 (s, 3 H, 32-H), 2.06 (m, 1 H, 39-H), 1.91 (ddd, $^2J_{16',16}$ = 14.3 Hz, $^3J_{16',10}$ = $^3J_{16',17}$ = 6.8 Hz, 1 H, 16'-H), 1.68 (m, 4 H, 17-H, 25-H, 42-H), 1.51 (m, 3 H, 16-H, 25'-H, 43-H), 1.40 (s, 9 H, 31-H), 1.42 (m, 2 H, 27-H), 1.17 (m, 2 H, 26-H), 0.97 (d, $^3J_{24,17}$ = 6.7 Hz, 3 H, 24-H), 0.91 (m, 9 H, 40-H, 44-H), 0.87 (d, $^3J_{44',43}$ = 5.7 Hz, 3 H, 44'-H).

^{13}**C-NMR** (125 MHz, DMSO-d$_6$, 373 K): δ = 171.1 (s, C-3), 170.8 (s, C-7), 170.8 (s, C-1), 169.6 (s, C-9), 168.0 (s, C-5), 155.3 (s, C-11), 154.4 (s, C-29), 138.3 (s, C-20), 137.6 (s, C-34), 132.9 (d, C-13), 127.5 (d, C-22), 127.3 (d, C-36), 126.9 (d, C-37), 126.7 (d, C-21), 126.6 (d, C-23), 126.5 (d, C-35), 116.3 (t, C-14), 81.7 (d, C-33), 77.7 (s, C-30), 74.2 (t, C-18), 71.8 (t, C-19), 65.0 (t, C-12), 57.0 (d, C-6), 56.4 (q, C-38), 56.4 (d, C-10), 54.2 (d, C-2), 53.3 (d, C-4), 52.4 (d, C-8), 51.1 (q, C-45), 47.5 (t, C-28), 36.4 (t, C-42), 33.2 (q, C-32), 31.9 (t, C-16), 30.9 (t, C-25), 30.8 (q, C-41), 30.1 (d, C-39), 29.7 (d, C-17), 29.5 (q, C-15), 27.7 (q, C-31), 26.5 (t, C-27), 23.9 (d, C-43), 22.2 (q, C-44), 22.0 (t, C-26), 21.0 (q, C-44‘), 18.5 (q, C-40), 17.1 (q, C-40‘), 17.0 (q, C-24).

optical rotation: $[\alpha]_D^{20}$ = –61.8 (c = 1.0, $CHCl_3$)

**HRMS (ESI):**	calculated	found
$C_{53}H_{83}N_6O_{12}$ $[M+H]^+$	995.6063	995.6076

Methyl *N*-(((2*S*,3*R*)-2-((*S*)-2-((2*S*,4*R*)-2-((*S*)-2-(((allyloxy)carbonyl)amino)-*N*-methyl-3-(1-methyl-1*H*-indol-3-yl)propanamido)-5-(benzyloxy)-4-methylpentanamido)-6-((*tert*-butoxycarbonyl)(methyl)amino)hexanamido)-3-methoxy-3-phenylpropanoyl)-L-valyl)-*N*-methyl-L-leucinate (7)

According to Kazmaier and Junk,[6] 1,3 dimethylbarbituric acid (1.17 g, 7.51 mmol, 3.0 eq.) and $Pd(PPh_3)_4$ (87.0 mg, 75.0 µmol, 3 mol%) were added to a solution of pentapeptide **6** (2.49 g, 2.51 mmol, 1.0 eq.) in 25 mL anhydrous DCM at room temperature. The reaction mixture was stirred for two hours at this temperature and then diluted with EtOAc. After washing three times with a sat. $NaHCO_3$ solution, the aqueous phase was extracted once with EtOAc. The combined organic phases were dried over Na_2SO_4 and concentrated under reduced pressure. Subsequently, the residue was dissolved in 50 mL anhydrous DCM, Alloc-Trp(*N*-Me)-OH (833 mg, 2.75 mmol, 1.1 eq.) was added and the mixture was cooled to –20 °C. After addition of NMM (ρ = 0.92 g/mL, 606 µL, 5.51 mmol, 2.2 eq.) and BEP (754 mg, 2.75 mmol, 1.1 eq.), the reaction mixture was slowly warmed to room temperature, diluted with DCM after 16 h (LC/MS control), washed successively with water, sat. $NaHCO_3$ solution and brine and dried over Na_2SO_4. Removal of the solvent under reduced pressure and purification by column chromatography (C18 spherical, H_2O/MeCN 9:1 to MeCN) yielded hexapeptide **7** (2.63 g, 2.20 mmol, 88%) as a yellowish foam.

LC/MS: t_R (7) = 1.75 min

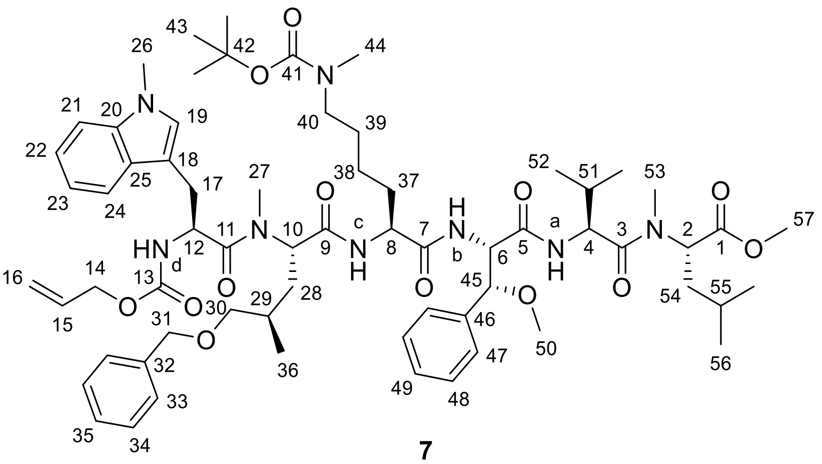

7

^{1}H-NMR (500 MHz, DMSO-d$_6$, 373 K): δ = 7.66 (s, 1 H, NH$_b$), 7.55 (d, $^3J_{24,23}$ = 7.8 Hz, 1 H, 24-H), 7.49 (d, $^3J_{NHa,4}$ = 7.2 Hz, 1 H, NH$_a$), 7.27 (m, 12 H, 21-H, 33-H, 34-H, 35-H, 47-H, 48-H, 49-H, NH$_c$), 7.13 (dd, $^3J_{22,21}$ = $^3J_{22,23}$ = 7.5 Hz, 1 H, 22-H), 7.07 (s, 1 H, 19-H), 7.01 (dd, $^3J_{23,22}$ = $^3J_{23,24}$ = 7.4 Hz, 1 H, 23-H), 5.83 (m, 1 H, 15-H), 5.21 (d, $^3J_{16,15}$ = 16.9 Hz, 1 H, 16-H), 5.12 (d, $^3J_{16',15}$ = 11.1 Hz, 1 H, 16'-H), 5.05 (m, 2 H, 2-H, 10-H), 4.74 (dt, $^3J_{12,NHd}$ ≈ $^3J_{12,17}$ = 7.1 Hz, 1 H, 12-H), 4.72 (m, 1 H, 45-H), 4.65 (dd, $^3J_{4,51}$ = $^3J_{4,NHa}$ = 8.0 Hz, 1 H, 4-H), 4.58 (m, 1 H, 6-H), 4.43 (m, 4 H, 14-H, 31-H), 4.31 (td, $^3J_{8,37}$ = 8.2 Hz, $^3J_{8,NHc}$ = 5.5 Hz, 1 H, 8-H), 3.70 (s, 3 H, 26-H), 3.63 (s, 3 H, 57-H), 3.37 (m, 1 H, 30-H), 3.27 (m, 1 H, 30'-H), 3.18 (s, 3 H, 50-H), 3.13 (m, 1 H, 17-H), 3.05 (t, $^3J_{40,39}$ = 7.2 Hz, 2 H, 40-H), 3.01 (m, 1 H, 17'-H), 2.92 (m, 6 H, 27-H, 53-H), 2.70 (s, 3 H, 44-H), 2.06 (m, 1 H, 51-H), 1.92 (m, 1 H, 28-H), 1.66 (m, 4 H, 29-H, 37-H, 54-H), 1.44 (m, 5 H, 28'-H, 37'-H, 39-H, 55-H), 1.38 (s, 9 H, 43-H), 1.14 (m, 2 H, 38-H), 0.96 (m, 3 H, 36-H), 0.92 (d, $^3J_{52,51}$ = 7.2 Hz, 3 H, 52-H), 0.90 (d, $^3J_{52',51}$ = $^3J_{56,55}$ = 6.9 Hz, 6 H, 52'-H, 56-H), 0.87 (d, $^3J_{56',55}$ = 6.7 Hz, 3 H, 56'-H).

^{13}C-NMR (125 MHz, DMSO-d$_6$, 373 K): δ = 171.9 (s, C-11), 171.1 (s, C-3), 170.8 (s, C-1), 170.7 (s, C-7), 169.4 (s, C-9), 168.0 (s, C-5), 155.0 (s, C-13), 154.3 (s, C-41), 138.4 (s, C-32), 137.6 (s, C-46), 136.3 (s, C-20), 132.9 (d, C-15), 127.6 (d, C-19), 127.5 (d, C-34), 127.3 (s, C-25), 127.3 (d, C-48), 126.9 (d, C-49), 126.7 (d, C-33), 126.6 (d, C-35), 126.5 (d, C-47), 120.5 (d, C-22), 118.0 (d, C-23), 117.7 (d, C-24), 116.3 (t, C-16), 108.9 (d, C-21), 108.8 (s, C-18), 81.7 (d, C-45), 77.7 (s, C-42), 74.1 (t, C-30), 71.7 (t, C-31), 64.0 (t, C-14), 57.0 (d, C-6), 56.4 (q, C-50), 54.5 (d, C-10), 54.2 (d, C-2), 53.3 (d, C-4), 52.4 (d, C-8), 51.5 (d, C-12), 51.1 (q, C-57), 47.5 (t, C-40), 36.4 (t, C-54), 33.2 (q, C-44), 31.6 (q, C-26), 31.1 (t, C-28, t, C-37), 30.8 (q, C-53), 30.2 (q, C-27), 30.0 (d, C-51), 29.4 (d, C-29), 27.6 (q, C-43), 26.8 (t, C-17), 26.5 (t, C-39), 23.9 (d, C-55), 22.2 (q, C-56), 22.0 (t, C-38), 21.0 (q, C-56'), 18.4 (q, C-52), 17.1 (q, C-52'), 17.0 (q, C-36).

**optical rotation:**	$[\alpha]_D^{20}$ = –51.6 (c = 1.0, $CHCl_3$)	
**HRMS (ESI):**	calculated	found
$C_{65}H_{95}N_8O_{13}$ [M+H]$^+$	1195.7013	1195.6986

Methyl *N*-(((2*S*,3*R*)-2-((*S*)-2-((2*S*,4*R*)-2-((*S*)-2-((*S*)-2-(((allyloxy)carbonyl)amino)-4-(*tert*-butoxy)-4-oxobutanamido)-*N*-methyl-3-(1-methyl-1*H*-indol-3-yl)propanamido)-5-(benzyloxy)-4-methylpentanamido)-6-((*tert*-butoxycarbonyl)(methyl)amino)hexan-amido)-3-methoxy-3-phenylpropanoyl)-L-valyl)-*N*-methyl-L-leucinate (8)

According to Kazmaier and Junk,[6] 1,3 dimethylbarbituric acid (19.6 mg, 125 µmol, 3.0 eq.) and $Pd(PPh_3)_4$ (1.5 mg, 1.3 µmol, 3 mol%) were added to a solution of hexapeptide **7** (50.0 mg, 42.0 µmol, 1.0 eq.) in 420 µL anhydrous DCM at room temperature. The reaction mixture was stirred for two hours at room temperature and then diluted with EtOAc. After washing three times with a sat. $NaHCO_3$ solution, the aqueous phase was extracted once with EtOAc. The combined organic phases were dried over Na_2SO_4 and concentrated under reduced pressure. Subsequently, the residue was dissolved in 420 µL anhydrous DCM and Alloc-Asp(O*t*-Bu)-OH (13.7 mg, 50.0 µmol, 1.2 eq.) was added. After cooled to 0 °C, DIPEA (ρ = 0.74 g/mL, 15.4 µL, 88.0 µmol, 2.1 eq.) and PyAOP (27.5 mg, 53.0 µmol, 1.26 eq.) were added and the reaction mixture was slowly warmed to room temperature (18 h, LC/MS control). The solvent was removed under reduced pressure and the residue was suspended in EtOAc. The organic phase was washed successively with 1 M $KHSO_{4aq}$, sat. $NaHCO_3$ solution and brine, dried over Na_2SO_4 and concentrated under reduced pressure. Purification by column chromatography (C18 spherical, H_2O/MeCN 9:1 to MeCN) yielded the heptapeptide **8** (51.0 mg, 37.0 µmol, 89%) as a yellowish foam.

LC/MS: t_R (8) = 1.77 min

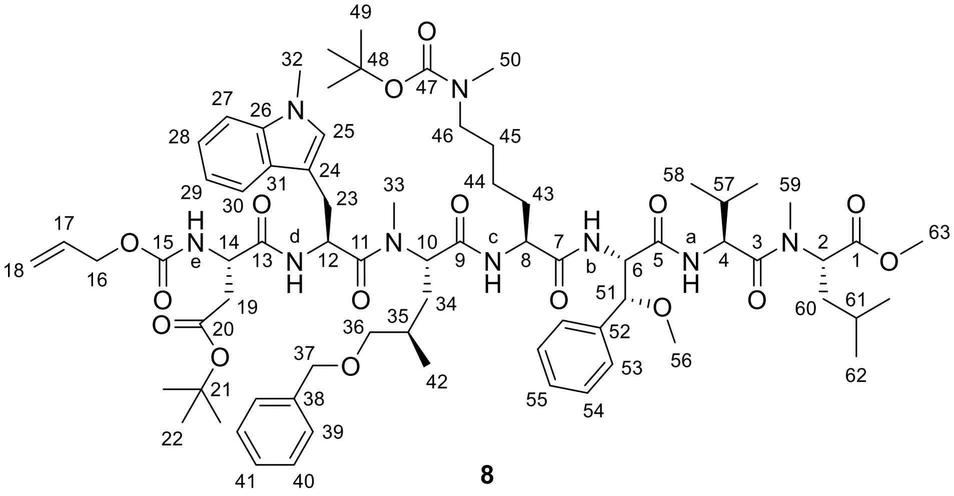

8

^{1}H-NMR (500 MHz, DMSO-d$_6$, 373 K): δ = 7.80 (s, 1 H, NH_d), 7.67 (s, 1 H, NH_b), 7.55 (d, $^3J_{30,29}$ = 7.0 Hz, 1 H, 30-H), 7.49 (d, $^3J_{NHa,4}$ = 7.3 Hz, 1 H, NH_a), 7.26 (m, 12 H, 27-H, 39-H, 40-H, 41-H, 53-H, 54-H, 55-H, NH_c), 7.12 (dd, $^3J_{28,27}$ = $^3J_{28,29}$ = 8.0 Hz, 1 H, 28-H), 7.04 (s, 1 H, 25-H), 7.01 (dd, $^3J_{29,28}$ = $^3J_{29,30}$ = 7.4 Hz, 1 H, 29-H), 6.96 (d, $^3J_{NHe,14}$ = 7.8 Hz, 1 H, NH_e), 5.89 (ddt, $^3J_{17,18}$ = 17.2 Hz, $^3J_{17,18'}$ = 10.7 Hz, $^3J_{17,16}$ = 5.3 Hz, 1 H, 17-H), 5.28 (dd, $^3J_{18,17}$ = 17.4 Hz, $^2J_{18,18'}$ = 1.5 Hz, 1 H, 18-H), 5.16 (dd, $^3J_{18',17}$ = 10.5 Hz, $^2J_{18',18}$ = 1.2 Hz, 1 H, 18'-H), 5.08 (m, 2 H, 10-H, 2-H), 4.99 (dt, $^3J_{12,23}$ = $^3J_{12,NHd}$ = 7.0 Hz, 1 H, 12-H), 4.71 (s, 1 H, 51-H), 4.64 (dd, $^3J_{4,57}$ = $^3J_{4,NHa}$ = 7.9 Hz, 1 H, 4-H), 4.60 (dd, $^3J_{6,NHb}$ = 8.5 Hz, $^3J_{6,51}$ = 3.2 Hz, 1 H, 6-H), 4.48 (m, 4 H, 16-H, 37-H), 4.39 (m, 1 H, 14-H), 4.28 (m, 1 H, 8-H), 3.69 (s, 3 H, 32-H), 3.63 (s, 3 H, 63-H), 3.36 (m, 1 H, 36-H), 3.27 (m, 1 H, 36'-H), 3.18 (s, 3 H, 56-H), 3.16 (m, 1 H, 23-H), 3.05 (m, 2 H, 46-H), 2.98 (m, 1 H, 23'-H), 2.93 (s, 3 H, 59-H), 2.84 (s, 3 H, 33-H), 2.70 (s, 3 H,

50-H), 2.59 (m, 1 H, 19-H), 2.43 (m, 1 H, 19‘-H), 2.05 (m, 1 H, 57-H), 1.93 (m, 1 H, 34-H), 1.66 (m, 4 H, 35-H, 43-H, 60-H), 1.44 (m, 5 H, 34‘-H, 43‘-H, 45-H, 61-H), 1.39 (s, 9 H, 22-H), 1.38 (s, 9 H, 49-H), 1.13 (m, 1 H, 44-H), 0.94 (m, 3 H, 42-H), 0.91 (d, $^3J_{58,57}$ = 6.7 Hz, 3 H, 58-H), 0.90 (d, $^3J_{58',57}$ = $^3J_{62,61}$ = 6.9 Hz, 6 H, 58‘-H, 62-H), 0.87 (m, 3 H, 62‘-H).

^{13}C-NMR (125 MHz, DMSO-d$_6$, 373 K): δ = 171.4 (s, C-11), 171.1 (s, C-3), 170.8 (s, C-1), 170.7 (s, C-7), 169.6 (s, C-13), 169.3 (s, C-9), 168.6 (s, C-20), 168.0 (s, C-5), 154.8 (s, C-15), 154.3 (s, C-47), 138.4 (s, C-38), 137.6 (s, C-52), 136.3 (s, C-26), 132.9 (d, C-17), 127.5 (d, C-25, d, C-40), 127.3 (s, C-31), 127.3 (d, C-54), 126.9 (d, C-55), 126.7 (d, C-39), 126.6 (d, C-41), 126.5 (d, C-53), 120.5 (d, C-28), 117.9 (d, C-29), 117.6 (d, C-30), 116.4 (t, C-18), 108.8 (d, C-27), 108.6 (s, C-24), 81.7 (d, C-51), 79.7 (s, C-21), 77.7 (s, C-48), 74.1 (t, C-36), 71.7 (t, C-37), 64.1 (t, C-16), 57.0 (d, C-6), 56.4 (q, C-56), 54.2 (d, C-2, d, C-10), 53.3 (d, C-4), 52.4 (d, C-8), 51.2 (d, C-14), 51.1 (q, C-63), 49.4 (d, C-12), 47.5 (t, C-46), 37.2 (t, C-19), 36.4 (t, C-60), 33.2 (q, C-50), 31.6 (q, C-32), 31.2 (t, C-34), 31.0 (t, C-43), 30.8 (q, C-59), 30.0 (d, C-57), 29.5 (d, C-35), 27.6 (q, C-49), 27.2 (q, C-22), 26.9 (t, C-23), 26.5 (t, C-45), 23.9 (d, C-61), 22.2 (q, C-62), 22.0 (t, C-44), 21.0 (q, C-62‘), 18.4 (q, C-58), 17.1 (q, C-58‘), 16.9 (q, C-42).

optical rotation: $[\alpha]_D^{20}$ = –48.2 (c = 1.0, $CHCl_3$)

**HRMS (ESI):**	calculated	found
$C_{73}H_{108}N_9O_{16}$ [M+H]$^+$	1366.7909	1366.7919

tert-Butyl 2-((2_S_,5_S_,8_S_,11_S_,14_S_,17_S_,20_S_)-8-((_R_)-3-(benzyloxy)-2-methylpropyl)-11-(4-((_tert_-butoxycarbonyl)(methyl)amino)butyl)-20-isobutyl-17-isopropyl-14-((_R_)-methoxy (phenyl)methyl)-7,19-dimethyl-5-((1-methyl-1_H_-indol-3-yl)methyl)-3,6,9,12,15,18,21-heptaoxo-1,4,7,10,13,16,19-heptaazacyclohenicosan-2-yl)acetate (9)

According to Nicolaou *et al.*,[7] trimethyltin hydroxide (258 mg, 1.43 mmol, 6.0 eq.) was added under nitrogen atmosphere to a solution of linear heptapeptide **8** (325 mg, 238 µmol, 1.0 eq.) in 2.4 mL anhydrous DCE and the reaction mixture was heated to 80 °C (6 h). Afterwards, the mixture was diluted with EtOAc. The organic phase was washed four times with 1 M $KHSO_{4aq}$ and once with brine, dried over Na_2SO_4 and concentrated under reduced pressure. After purification by column chromatography (C18 spherical, H_2O/MeCN 9:1 to MeCN), the desired acid (190 mg, 140 µmol, 59%) was isolated.

According to Genêt *et al.*,[8] the acid was dissolved in 1.4 mL of MeCN and water, followed by the addition of diethylamine (ρ = 0.71 g/mL, 73.4 µL, 702 µmol 5.0 eq.), TPPTS (3.2 mg, 5.6 µmol, 4 mol%) and $Pd(OAc)_2$ in MeCN (140 µL, 2.81 µmol, 0.02 M, 2 mol%) at room temperature. After stirring for 30 min, the solvent was removed under reduced pressure and the residue was dissolved in 4.5 mL anhydrous DMF.

In a three-necked flask, HATU (187 mg, 491 µmol, 3.5 eq.) and DIPEA (ρ = 0.74 g/mL, 110 µL, 631 µmol, 4.5 eq.) were dissolved in 136 mL anhydrous DCM at room temperature. Using a syringe pump, the previously dissolved free heptapeptide was added dropwise to the reaction mixture over six hours and stirred for additional 18 h (LC/MS control). The solvent was removed and the residue was dissolved in EtOAc. The organic phase was washed once with

1 M $KHSO_{4aq}$, three times with water and once with sat. $NaHCO_3$ solution and brine. After drying over Na_2SO_4, the solvent was removed under reduced pressure and the crude product was purified by column chromatography (C18 spherical, H_2O/MeCN 9:1 to MeCN) to obtain monocycle **9** (148 mg, 118 µmol, 50%) as a yellowish foam.

LC/MS: t_R (9) = 1.72 min

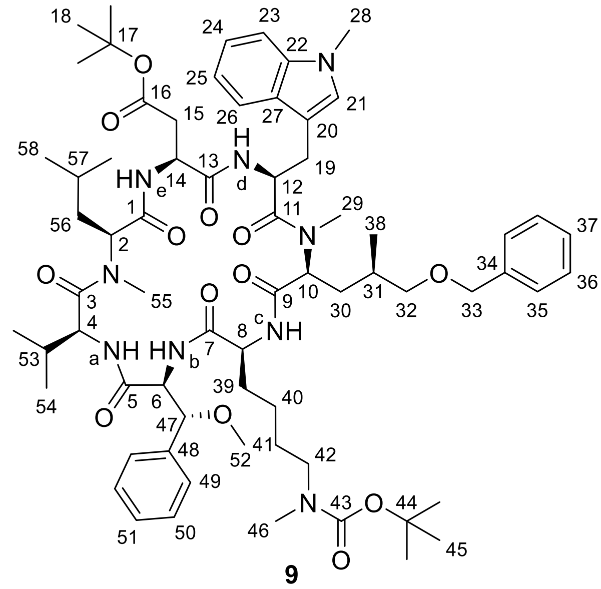

9

^{1}H-NMR (400 MHz, DMSO-d$_6$): δ = 9.38 (s, 1 H, NH_d), 9.15 (d, $^3J_{NHa,4}$ = 8.2 Hz, 1 H, NH_a), 8.53 (d, $^3J_{NHc,8}$ = 10.2 Hz, 1 H, NH_c), 8.02 (d, $^3J_{NHe,14}$ = 9.1 Hz, 1 H, NH_e), 7.48 (d, $^3J_{NHb,6}$ = 8.6 Hz, 1 H, NH_b), 7.39 (d, $^3J_{26,25}$ = 8.1 Hz, 1 H, 26-H), 7.22 (m, 12 H, 23-H, 24-H, 35-H, 36-H, 37-H, 49-H, 50-H, 51-H), 7.07 (s, 1 H, 21-H), 6.97 (dd, $^3J_{25,24}$ = $^3J_{25,26}$ = 7.5 Hz, 1 H, 25-H), 4.89 (d, $^3J_{47,6}$ = 2.7 Hz, 1 H, 47-H), 4.94 (m, 3 H, 2-H, 6-H, 14-H), 4.58 (m, 2 H, 10-H, 12-H), 4.47 (m, 1 H, 4-H), 4.16 (d, $^2J_{33,33'}$ = 12.2 Hz, 1 H, 33-H), 4.11 (d, $^2J_{33',33}$ = 12.1 Hz, 1 H, 33'-H), 4.01 (m, 1 H, 8-H), 3.65 (s, 3 H, 28-H), 3.13 (m, 2 H, 19-H), 2.99 (s, 3 H, 52-H), 3.03 (m, 2 H, 42-H), 2.93 (m, 1 H, 15-H), 2.70 (s, 6 H, 46-H, 55-H), 2.70 (m, 2 H, 32-H), 2.54 (m, 1 H, 15'-H), 2.45 (s, 3 H, 29-H), 2.22 (m, 2 H, 53-H, 56-H), 1.65 (m, 1 H, 30-H), 1.55 (m, 1 H, 57-H), 1.44 (s, 9 H, 18-H), 1.36 (s, 9 H, 45-H), 1.29 (m, 4 H, 31-H, 39-H, 41-H), 1.08 (m, 1 H, 39'-H), 0.98 (d, $^3J_{54,53}$ = 6.6 Hz, 3 H, 54-H), 1.00 (m, 1 H, 56'-H), 0.89 (d, $^3J_{58,57}$ = 6.9 Hz, 3 H, 58-H), 0.87 (d, $^3J_{58',57}$ = 7.1 Hz, 3 H, 58'-H), 0.84 (d, $^3J_{54',53}$ = 6.6 Hz, 3 H, 54'-H), 0.76 (m, 2 H, 40-H), 0.26 (d, $^3J_{38,31}$ = 6.5 Hz, 3 H, 38-H), –0.27 (m, 1 H, 30'-H).

^{13}C-NMR (100 MHz, DMSO-d$_6$): δ = 171.2 (s, C-13), 171.1 (s, C-3), 171.1 (s, C-11), 170.4 (s, C-5), 170.0 (s, C-7), 168.9 (s, C-16), 168.5 (s, C-9), 167.2 (s, C-1), 154.7 (s, C-43), 138.4 (s, C-34), 136.7 (s, C-48), 136.4 (s, C-22), 127.9 (d, C-21), 127.5 (s, C-27), 126.9–128.2 (d, C-35, C-36, C-37, C-49, C-50, C-51), 121.3 (d, C-24), 118.5 (d, C-25), 118.4 (d, C-26), 109.6 (d, C-23), 108.2 (s, C-20), 83.9 (d, C-47), 80.2 (s, C-17), 78.2 (s, C-44), 74.8 (t, C-32), 71.7 (t, C-33), 58.0 (d, C-2), 57.6 (d, C-10), 56.8 (q, C-52), 55.5 (d, C-6), 54.7 (d, C-4), 54.4 (d, C-8), 50.9 (d, C-12), 48.9 (d, C-14), 47.8 (t, C-42), 38.8 (t, C-56), 36.2 (t, C-15), 34.0 (t, C-39), 33.5 (q, C-46), 32.3 (q, C-28), 32.0 (t, C-30), 29.2 (d, C-31), 29.2 (d, C-53), 29.1 (q, C-29), 28.5 (q, C-55), 28.0 (q, C-45), 27.6 (q, C-18), 27.5 (t, C-19), 26.8 (t, C-41), 24.7 (d, C-57), 23.4 (q, C-58), 22.8 (t, C-40), 21.8 (q, C-58'), 19.6 (q, C-54), 19.1 (q, C-54'), 16.0 (q, C-38).

optical rotation: $[\alpha]_D^{20}$ = –82.5 (c = 1.0, $CHCl_3$)

**HRMS (ESI):**	calculated	Gefunden
$C_{68}H_{100}N_9O_{13}$ [M+H]$^+$	1250.7435	1250.7433

(1*S*,4*S*,7*S*,10*S*,13*S*,16*S*,19*S*)-16-((*R*)-3-(Benzyloxy)-2-methylpropyl)-4-isobutyl-7-isopropyl-10-((*R*)-methoxy(phenyl)methyl)-5,17,24-trimethyl-19-((1-methyl-1*H*-indol-3-yl) methyl)-2,5,8,11,14,17,20,24-octaazabicyclo[11.8.7]octacosan-3,6,9,12,15,18,21,23-octaone (10)

To a solution of cyclic heptapeptide **9** (100 mg, 80.0 µmol, 1.0 eq.) in 400 µL DCM, 400 µL TFA was added at room temperature and the mixture was stirred for two hours. The solvent was removed under reduced pressure and the residue was suspended three times in chloroform and concentrated each time under reduced pressure. After drying in high vacuum, the crude product was dissolved in 2.5 mL anhydrous DMF.

In a three-necked flask, HATU (107 mg, 281 µmol, 3.5 eq.) and DIPEA (ρ = 0.74 g/mL, 77.0 µL, 441 µmol, 5.5 eq.) were dissolved in 78 mL anhydrous DCM at room temperature. Using a syringe pump, the previously dissolved free monocycle was added dropwise to the reaction mixture over six hours and stirred for additional 17 h (LC/MS control). The solvent was removed and the residue was dissolved in EtOAc. The organic phase was washed once with 1 M $KHSO_{4aq}$, three times with water and once with sat. $NaHCO_3$ solution and brine. After drying over Na_2SO_4, the solvent was removed under reduced pressure and the crude product was purified by column chromatography (C18 spherical, H_2O/MeCN 9:1 to MeCN) to obtain the protected bicycle **10** (31.0 mg, 29.0 µmol, 36%) as a yellowish foam.

LC/MS: t_R (10) = 1.49 min

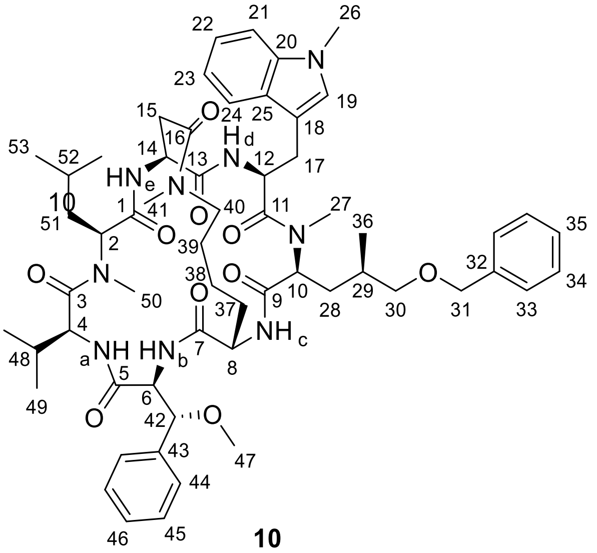

10

^{1}H-NMR (500 MHz, DMSO-d$_6$): δ = 9.32 (d, $^3J_{NHd,12}$ = 4.3 Hz, 1 H, NH$_d$), 9.13 (d, $^3J_{NHa,4}$ = 7.8 Hz, 1 H, NH$_a$), 8.78 (d, $^3J_{NHc,8}$ = 10.4 Hz, 1 H, NH$_c$), 8.31 (d, $^3J_{NHe,14}$ = 9.3 Hz, 1 H, NH$_e$), 7.36 (d, $^3J_{24/NHb,23/6}$ = 7.9 Hz, 2 H, 24-H, NH$_b$), 7.27 (m, 11 H, 21-H, 33-H, 34-H, 35-H, 44-H, 45-H, 46-H), 7.08 (dd, $^3J_{22,21}$ = $^3J_{22,23}$ = 7.3 Hz, 1 H, 22-H), 7.02 (s, 1 H, 19-H), 6.95 (dd, $^3J_{23,22}$ = $^3J_{23,24}$ = 7.2 Hz, 1 H, 23-H), 5.17 (ddd, $^3J_{14,15}$ = 10.8 Hz, $^3J_{14,NHe}$ = 9.6 Hz, $^3J_{14,15'}$ = 4.3 Hz, 1 H, 14-H),

4.98 (dd, $^{3}J_{2,51}$ = 10.7 Hz, $^{3}J_{2,51'}$ = 3.0 Hz, 1 H, 2-H), 4.90 (d, $^{3}J_{42,6}$ = 2.1 Hz, 1 H, 42-H), 4.81 (dd, $^{3}J_{6,NHb}$ = 6.9 Hz, $^{3}J_{6,42}$ = 2.4 Hz, 1 H, 6-H), 4.50 (m, 3 H, 4-H, 10-H, 12-H), 4.25 (m, 1 H, 8-H), 4.23 (d, $^{2}J_{31,31'}$ = 12.4 Hz, 1 H, 31-H), 4.18 (d, $^{2}J_{31',31}$ = 12.1 Hz, 1 H, 31‘-H), 3.62 (s, 3 H, 26-H), 3.38 (m, 1 H, 40-H), 3.13 (s, 3 H, 47-H), 3.16 (m, 1 H, 17-H), 3.06 (m, 2 H, 17‘-H, 40‘-H), 2.95 (m, 1 H, 15-H), 2.77 (s, 3 H, 41-H), 2.74 (s, 3 H, 50-H), 2.67 (m, 1 H, 30-H), 2.63 (dd, $^{2}J_{30',30}$ = 6.8 Hz, $^{3}J_{30',29}$ = 1.9 Hz, 1 H, 30‘-H), 2.32 (s, 3 H, 27-H), 2.25 (m, 3 H, 15‘-H, 48-H, 51-H), 1.53 (m, 3 H, 28-H, 39-H, 52-H), 1.31 (m, 3 H, 37-H, 38-H, 39‘-H), 1.16 (m, 3 H, 29-H, 37‘-H, 38‘-H), 1.01 (d, $^{3}J_{49,48}$ = 6.6 Hz, 3 H, 49-H), 0.94 (d, $^{3}J_{53,52}$ = 6.6 Hz, 3 H, 53-H), 0.93 (d, $^{3}J_{53',52}$ = 6.4 Hz, 3 H, 53‘-H), 0.87 (d, $^{3}J_{49',48}$ = 6.6 Hz, 3 H, 49‘-H), 0.17 (d, $^{3}J_{36,29}$ = 6.7 Hz, 3 H, 36-H), –0.75 (m, 1 H, 28‘-H).

^{13}C-NMR (125 MHz, DMSO-d$_6$): δ = 171.3 (s, C-13), 171.3 (s, C-11), 170.8 (s, C-3), 170.1 (s, C-5), 168.8 (s, C-7), 168.0 (s, C-16), 167.7 (s, C-9), 167.0 (s, C-1), 138.4 (s, C-32), 137.1 (s, C-43), 136.3 (s, C-20), 128.3 (d, C-19), 127.7 (d, C-33), 127.4 (s, C-25), 127.2–128.2 (d, C-34, C-35, C-45, C-46), 126.6 (d, C-44), 121.2 (d, C-22), 118.5 (d, C-23), 118.3 (d, C-24), 109.6 (d, C-21), 108.3 (s, C-18), 83.3 (d, C-42), 75.0 (t, C-30), 71.7 (t, C-31), 57.9 (d, C-2), 57.5 (d, C-10), 57.2 (q, C-47), 56.5 (d, C-6), 54.9 (d, C-4), 53.8 (d, C-8), 51.1 (d, C-12), 50.5 (d, C-14), 49.1 (t, C-40), 39.0 (t, C-51), 34.6 (t, C-15), 32.2 (q, C-26), 32.2 (q, C-41), 31.5 (t, C-28), 31.2 (t, C-37), 29.3 (d, C-48), 28.9 (d, C-29), 28.7 (q, C-27), 28.5 (q, C-50), 27.4 (t, C-17), 26.8 (t, C-39), 24.7 (d, C-52), 23.4 (q, C-53), 22.0 (q, C-53‘), 21.2 (t, C-38), 19.6 (q, C-49), 19.0 (q, C-49‘), 15.7 (q, C-36).

**optical rotation:**	$[\alpha]_D^{20}$ = –43.8 (c = 0.5, CHCl$_3$)	
**HRMS (ESI):**	calculated	found
$C_{59}H_{82}N_9O_{10}$ [M+H]$^+$	1076.6179	1076.6199

(1*S*,4*S*,7*S*,10*S*,13*S*,16*S*,19*S*)-16-((*R*)-3-Hydroxy-2-methylpropyl)-4-isobutyl-7-isopropyl-10-((*R*)-methoxy(phenyl)methyl)-5,17,24-trimethyl-19-((1-methyl-1*H*-indol-3-yl)methyl)-2,5,8,11,14,17,20,24-octaazabicyclo[11.8.7]octacosan-3,6,9,12,15,18,21,23-octaone (1)

According to Panek and Xu,[9] boron trichloride in heptane (ρ = 0.74 g/mL, 20.5 µL, 21.0 µmol, 1.0 M, 1.7 eq.) was added dropwise to a solution of the protected bicyclic **10** (13.0 mg, 12.0 µmol, 1.0 eq.) in 120 µL anhydrous DCM under nitrogen atmosphere at –78 °C. After one hour, boron trichloride in heptane (ρ = 0.74 g/mL, 20.5 µL, 21.0 µmol, 1.0 M, 1.7 eq.) was added again and the reaction mixture was warmed to –50 °C within one hour. Due to incomplete conversion, the reaction mixture was cooled to –78 °C and boron trichloride in heptane (ρ = 0.74 g/mL, 41.1 µL, 41.0 µmol, 1.0 M, 3.4 eq.) was added again. Subsequently, the reaction mixture was slowly warmed to –25 °C (2 h, LC/MS control) and hydrolyzed with 40 µL methanol. After stirring for 30 min at room temperature, the solvent was removed under reduced pressure and the crude product was purified by column chromatography (C18 spherical, H_2O/MeCN 9:1 to MeCN) and preparative HPLC (Luna®, H_2O/MeCN 9:1 to H_2O/MeCN 5:95) to obtain the bicyclic cyclomarin derivative **1** (1.5 mg, 1.5 µmol, 13%) as a colorless amorphous solid.

LC/MS: t$_R$ (1) = 1.18 min

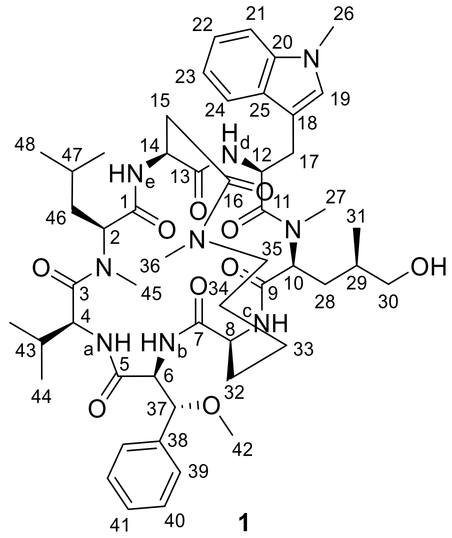

1

^{1}H-NMR (500 MHz, $CDCl_3$): δ = 8.59 (d, $^{3}J_{NHe,14}$ = 9.2 Hz, 1 H, NH_e), 8.42 (d, $^{3}J_{NHa,4}$ = 8.2 Hz, 1 H, NH_a), 8.26 (d, $^{3}J_{NHc,8}$ = 10.8 Hz, 1 H, NH_c), 7.48 (d, $^{3}J_{24,23}$ = 7.9 Hz, 1 H, 24-H), 7.25 (m, 9 H, 21-H, 22-H, 39-H, 40-H, 41-H, NH_b, NH_d), 7.08 (dd, $^{3}J_{23,22}$ = $^{3}J_{23,24}$ = 7.0 Hz, 1 H, 23-H), 6.81 (s, 1 H, 19-H), 5.49 (d, $^{3}J_{37,6}$ = 6.0 Hz, 1 H, 37-H), 5.25 (ddd, $^{3}J_{14,15}$ = 11.2 Hz, $^{3}J_{14,NHe}$ = 9.2 Hz, $^{3}J_{14,15'}$ = 4.7 Hz, 1 H, 14-H), 4.91 (dd, $^{3}J_{2,46}$ = 10.5 Hz, $^{3}J_{2,46'}$ = 3.3 Hz, 1 H, 2-H), 4.86 (m, 3 H, 6-H, 8-H, 12-H), 4.61 (dd, $^{3}J_{4,43}$ = $^{3}J_{4,NHa}$ = 8.6 Hz, 1 H, 4-H), 4.33 (dd, $^{3}J_{10,28}$ = 11.0 Hz, $^{3}J_{10,28'}$ = 2.1 Hz, 1 H, 10-H), 3.70 (s, 3 H, 26-H), 3.40 (dd, $^{2}J_{17,17'}$ = 13.4 Hz, $^{3}J_{17,12}$ = 11.0 Hz, 1 H, 17-H), 3.29 (s, 3 H, 42-H), 3.12 (dd, $^{2}J_{17',17}$ = 13.6 Hz, $^{3}J_{17',12}$ = 4.4 Hz, 1 H, 17‘-H), 3.00 (m, 1 H, 30-H), 2.94 (s, 3 H, 45-H), 2.92 (m, 1 H, 30‘-H), 2.84 (s, 3 H, 36-H), 2.80 (m, 2 H, 35-H), 2.45 (s, 3 H, 27-H), 2.41 (ddd, $^{2}J_{46,46'}$ = 13.3 Hz, $^{3}J_{46,2}$ = 10.8 Hz, $^{3}J_{46,47}$ = 4.4 Hz, 1 H, 46-H), 2.25 (m, 1 H, 43-H), 2.13 (dd, $^{2}J_{15',15}$ = 14.7 Hz, $^{3}J_{15',14}$ = 4.7 Hz, 1 H, 15‘-H), 1.95 (dd, $^{2}J_{15,15'}$ = 14.7 Hz, $^{3}J_{15,14}$ = 11.5 Hz, 1 H, 15-H), 1.76 (ddd, $^{2}J_{28,28'}$ = 13.3 Hz, $^{3}J_{28,10}$ = 11.4 Hz, $^{3}J_{28,29}$ = 7.4 Hz, 1 H, 28-H), 1.58 (m, 2 H, 32-H, 47-H), 1.25 (m, 2 H, 32‘-H, 34-H), 1.15 (ddd, $^{2}J_{46',46}$ = 13.3 Hz, $^{3}J_{46',47}$ = 8.9 Hz, $^{3}J_{46',2}$ = 3.5 Hz, 1 H, 46‘-H), 1.10 (d, $^{3}J_{44,43}$ = 6.6 Hz, 3 H, 44-H), 1.05 (m, 1 H, 34‘-H), 1.02 (d, $^{3}J_{48,47}$ = 6.6 Hz, 3 H, 48-H), 1.01 (d, $^{3}J_{48',47}$ = 6.3 Hz, 3 H, 48‘-H), 1.00 (d, $^{3}J_{44',43}$ = 5.8 Hz, 3 H, 44‘-H), 0.72 (m, 2 H, 29-H, 33-H), 0.24 (d, $^{3}J_{31,29}$ = 6.9 Hz, 3 H, 31-H), 0.14 (m, 1 H, 33‘-H), –0.68 (ddd, $^{2}J_{28',28}$ = 13.3 Hz, $^{3}J_{28',29}$ = 6.6 Hz, $^{3}J_{28',10}$ = 2.2 Hz, 1 H, 28‘-H).

^{13}C-NMR (125 MHz, $CDCl_3$): δ = 172.7 (s, C-13), 171.1 (s, C-11), 170.5 (s, C-3), 170.2 (s, C-5, s, C-7), 169.3 (s, C-9), 168.3 (s, C-16), 167.8 (s, C-1), 136.8 (s, C-20), 136.3 (s, C-38), 128.9 (d, C-40), 128.8 (d, C-41), 128.4 (d, C-39), 127.7 (d, C-19), 127.5 (s, C-25), 122.2 (d, C-22), 119.5 (d, C-23), 118.7 (d, C-24), 109.6 (d, C-21), 108.2 (s, C-18), 79.8 (d, C-37), 65.7 (t, C-30), 59.2 (d, C-10), 58.9 (d, C-2), 57.5 (q, C-42), 56.5 (d, C-6), 55.4 (d, C-4), 54.4 (d, C-8), 50.7 (d, C-14), 50.3 (d, C-12), 48.7 (t, C-35), 39.2 (t, C-46), 36.3 (t, C-15), 32.9 (d, C-29, q, C-36), 32.7 (q, C-26), 32.6 (t, C-32), 31.1 (t, C-28), 30.6 (d, C-43), 29.0 (q, C-45), 28.9 (q, C-27), 28.4 (t, C-17), 27.2 (t, C-34), 25.2 (d, C-47), 23.6 (q, C-48), 22.5 (q, C-48‘), 20.8 (t, C-33), 20.0 (q, C-44), 19.3 (q, C-44‘), 17.1 (q, C-31).

**optical rotation:**	$[\alpha]_D^{20}$ = –22.7 (c = 0.15, $CHCl_3$)	
**HRMS (ESI):**	calculated	found
$C_{52}H_{76}N_9O_{10}$ $[M+H]^+$	986.5710	986.5726

Copies of the NMR spectra

Methyl *N*-(((2*S*,3*R*)-2-((*S*)-2-(((benzyloxy)carbonyl)amino)-6-((*tert*-butoxycarbonyl)(methyl)amino)hexanamido)-3-methoxy-3-phenylpropanoyl)-L-valyl)-*N*-methyl-L-leucinate (4)

^{1}H-NMR (500 MHz, DMSO-d$_6$, 373 K):

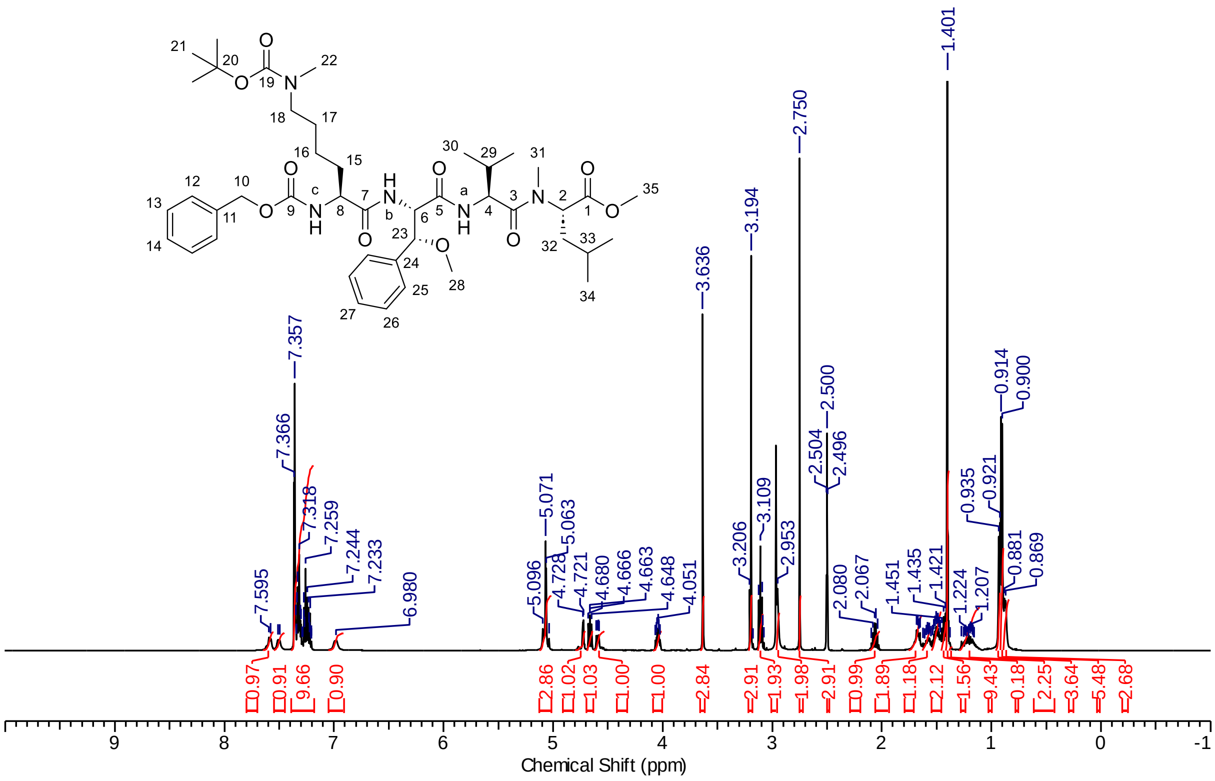

^{13}C-NMR (125 MHz, DMSO-d$_6$, 373 K):

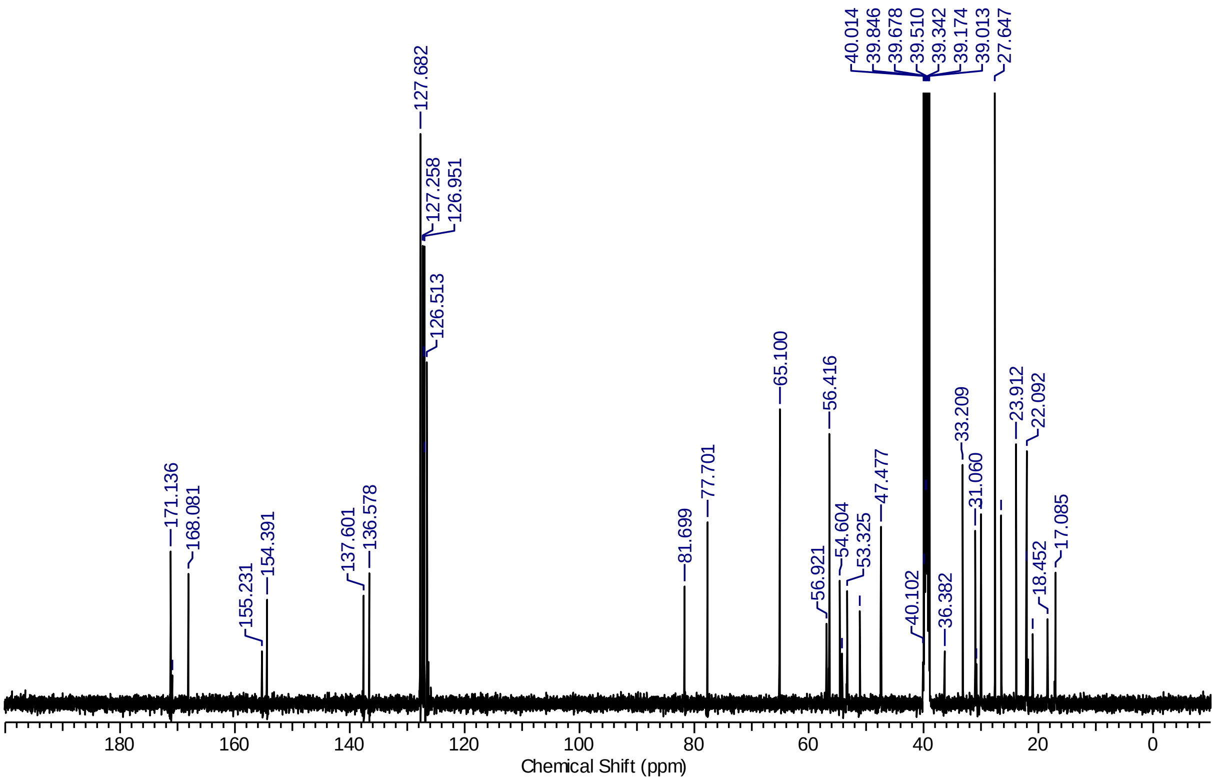

(2*S*,4*R*)-2-(((Allyloxy)carbonyl)(methyl)amino)-5-(benzyloxy)-4-methylpentanoic acid (5)

[1]**H-NMR** (500 MHz, $CDCl_3$):

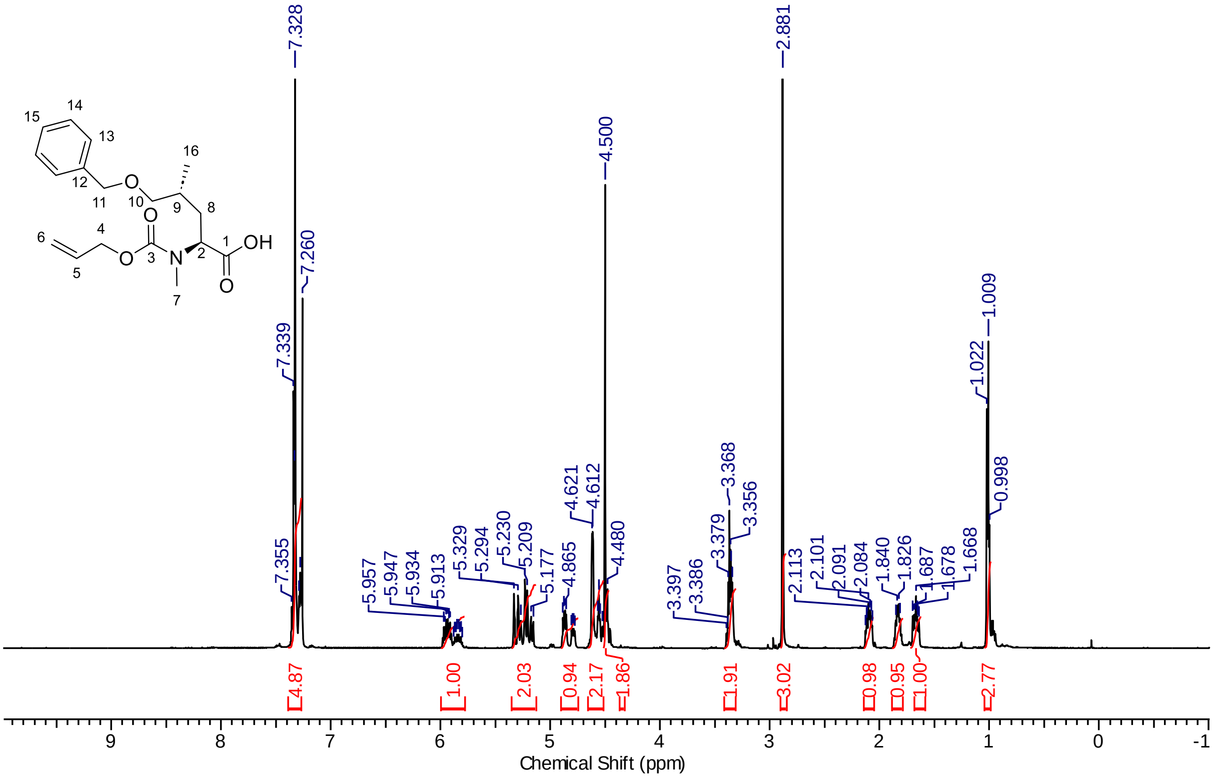

[13]**C-NMR** (100 MHz, $CDCl_3$):

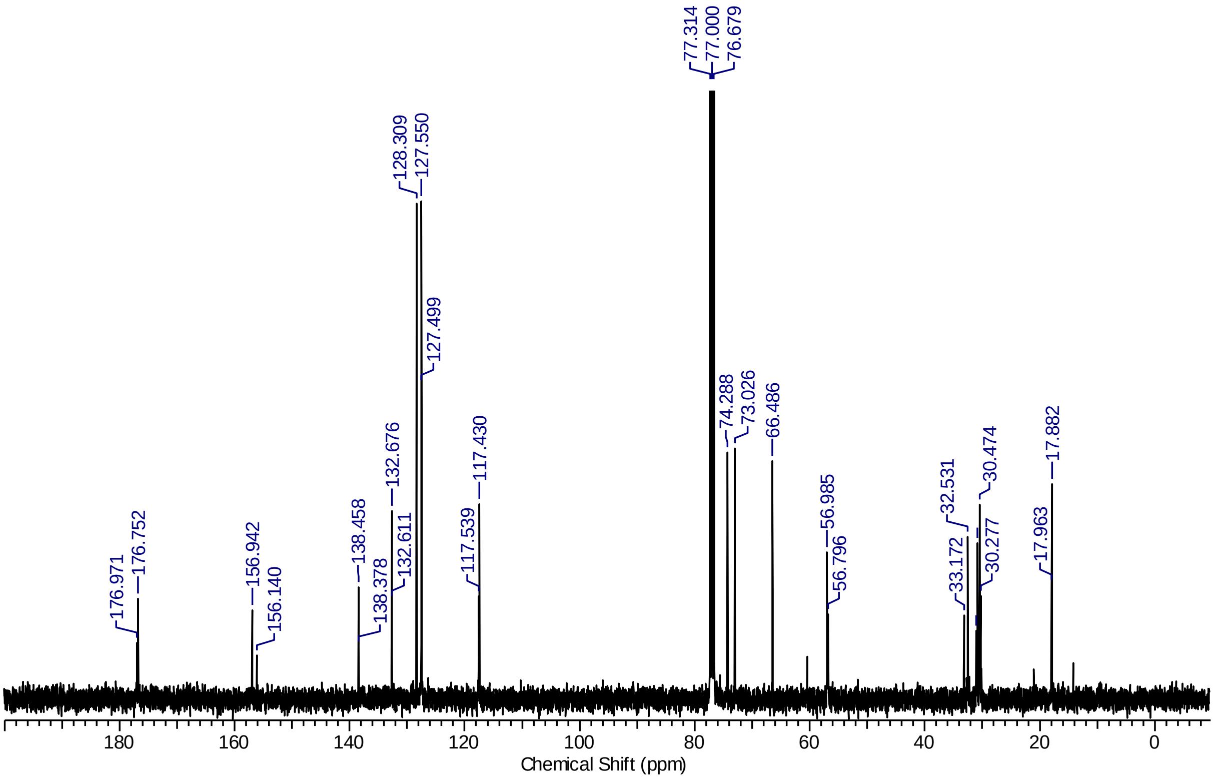

Methyl *N*-(((2*S*,3*R*)-2-((*S*)-2-((2*S*,4*R*)-2-(((allyloxy)carbonyl)(methyl)amino)-5-(benzyl-oxy)-4-methylpentanamido)-6-((*tert*-butoxycarbonyl)(methyl)amino)hexanamido)-3-methoxy-3-phenylpropanoyl)-L-valyl)-*N*-methyl-L-leucinate (6)

^{1}H-NMR (500 MHz, DMSO-d$_6$, 373 K):

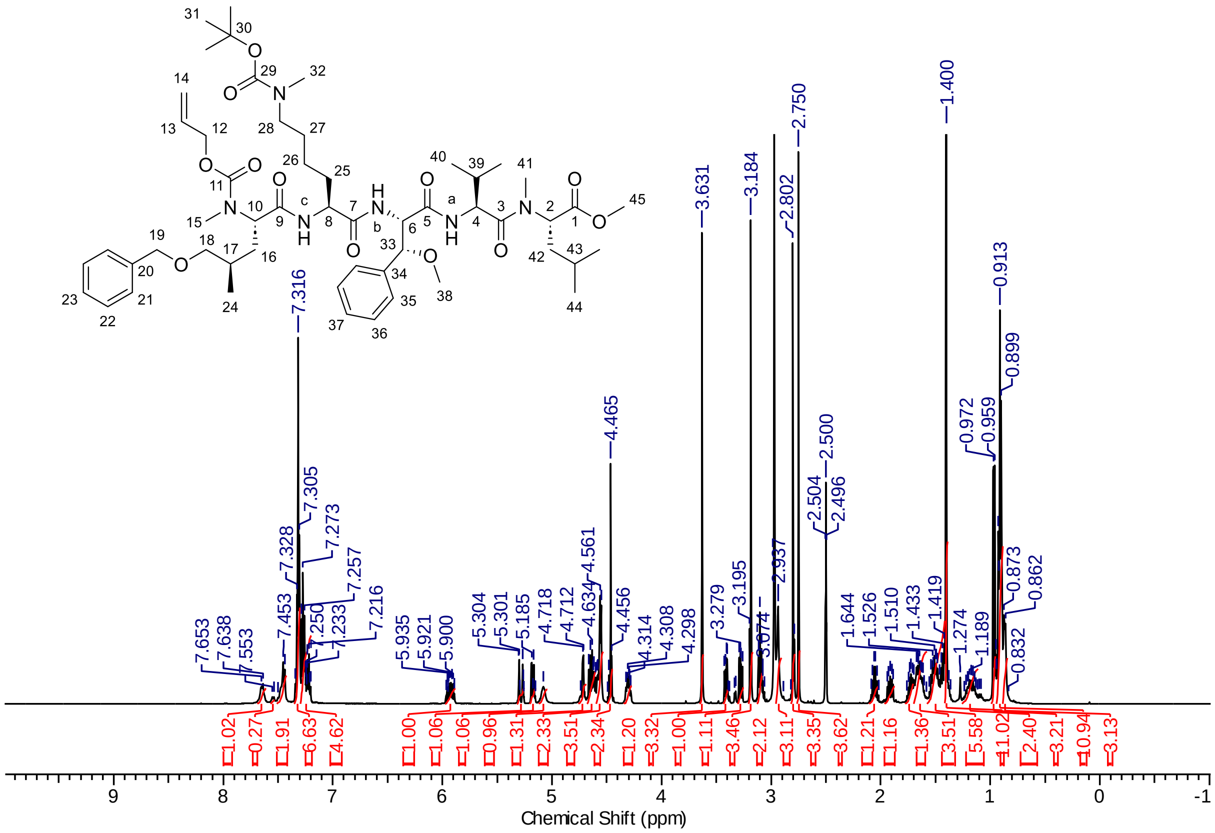

^{13}C-NMR (125 MHz, DMSO-d$_6$, 373 K):

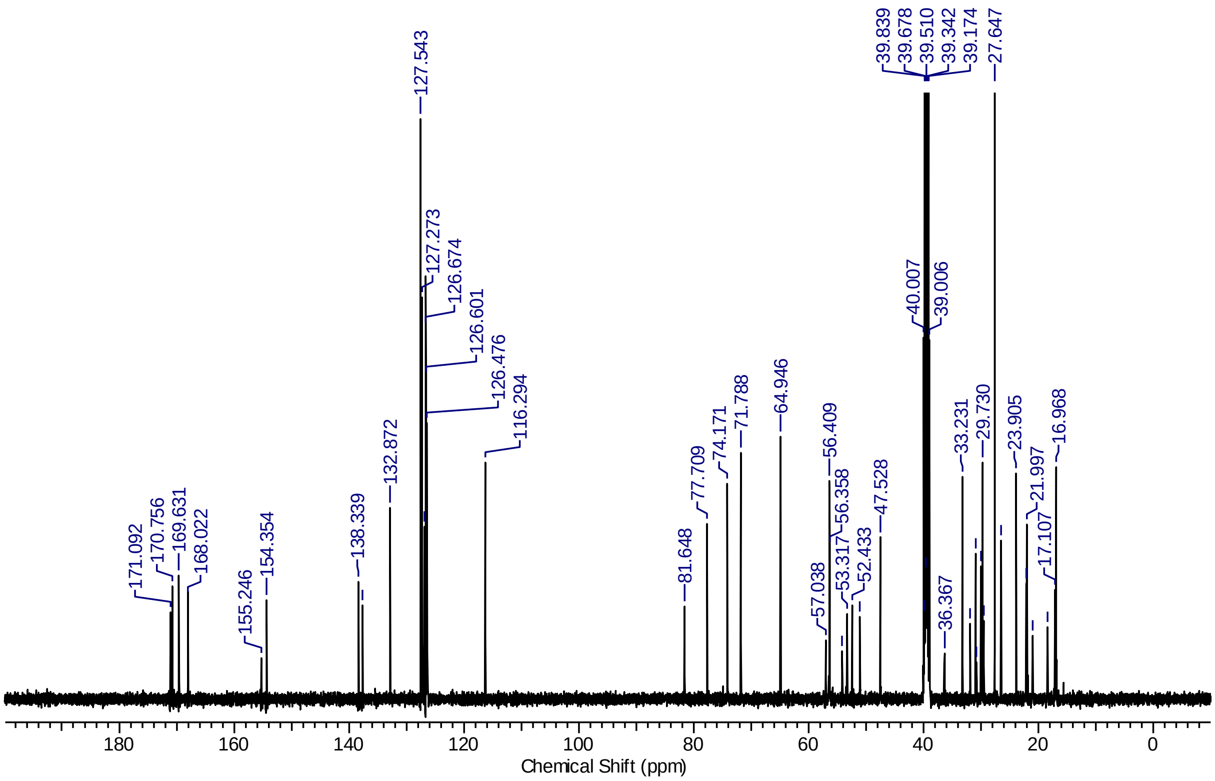

Methyl *N*-(((2*S*,3*R*)-2-((*S*)-2-((2*S*,4*R*)-2-((*S*)-2-(((allyloxy)carbonyl)amino)-*N*-methyl-3-(1-methyl-1*H*-indol-3-yl)propanamido)-5-(benzyloxy)-4-methylpentanamido)-6-((*tert*-butoxycarbonyl)(methyl)amino)hexanamido)-3-methoxy-3-phenylpropanoyl)-L-valyl)-*N*-methyl-L-leucinate (7)

^{1}H-NMR (500 MHz, DMSO-d$_6$, 373 K):

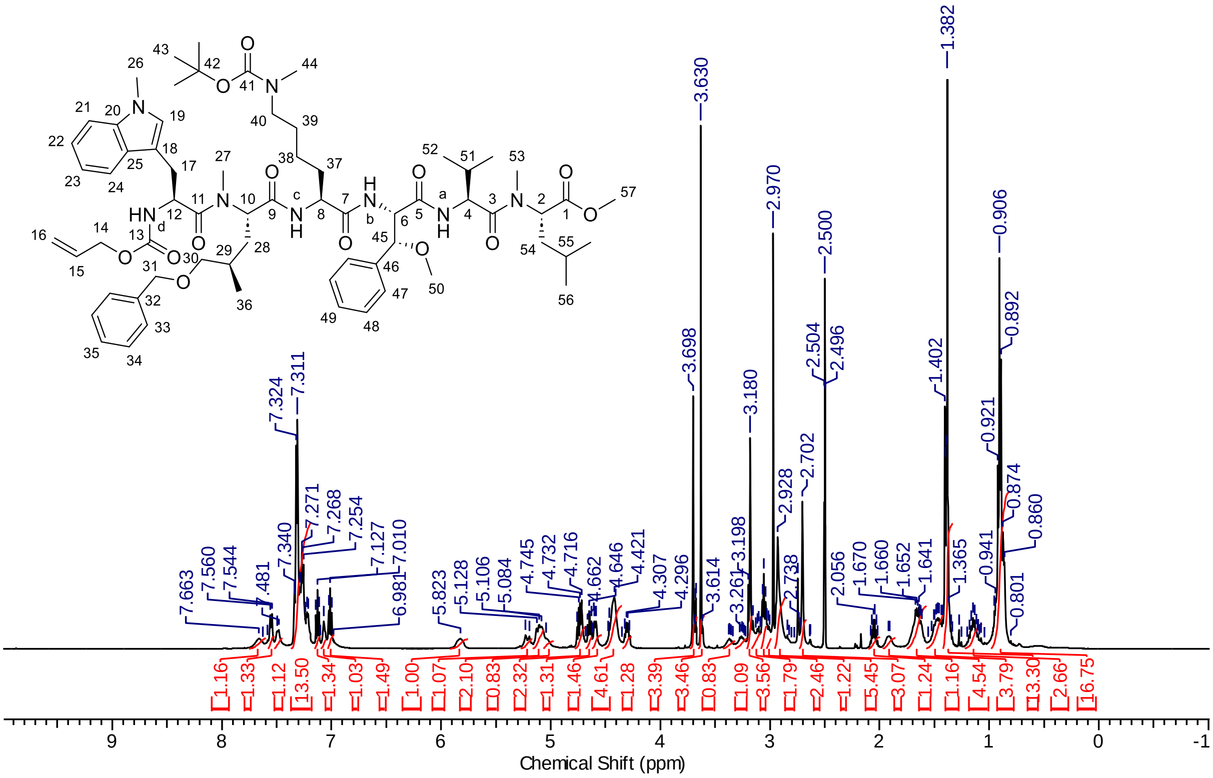

^{13}C-NMR (125 MHz, DMSO-d$_6$, 373 K):

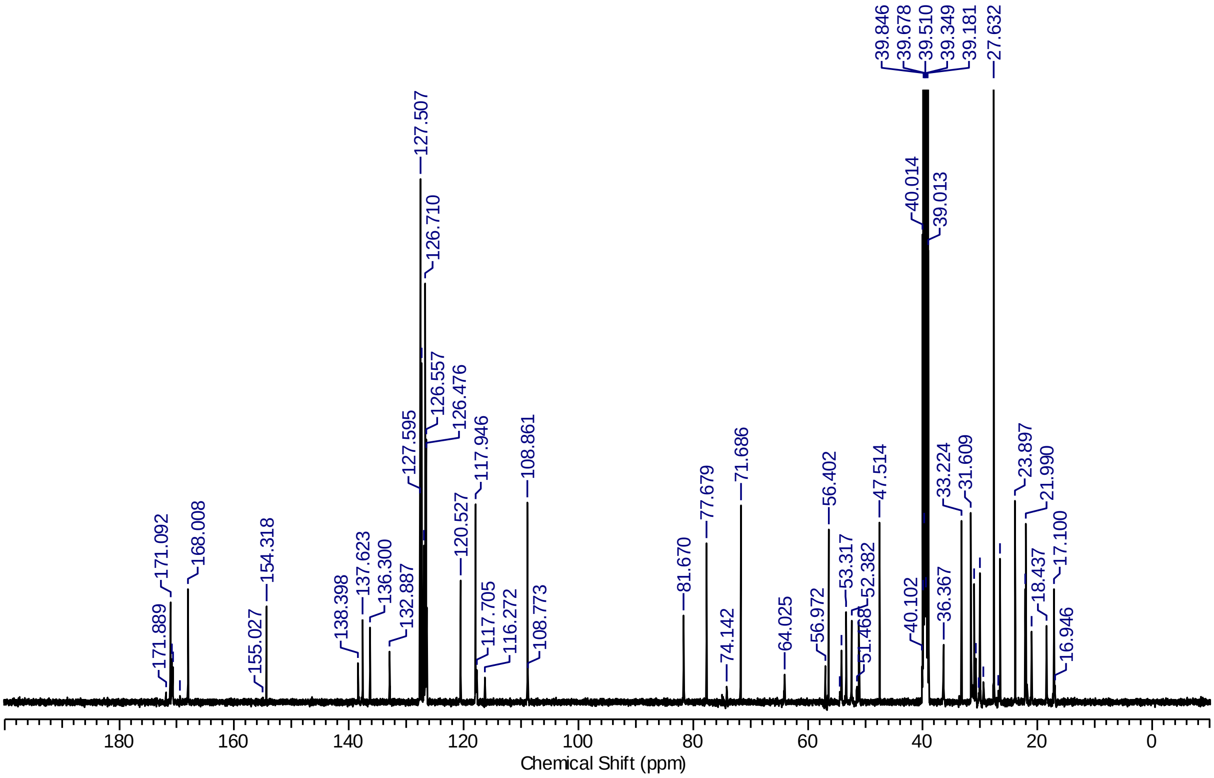

Methyl *N*-(((2*S*,3*R*)-2-((*S*)-2-((2*S*,4*R*)-2-((*S*)-2-((*S*)-2-(((allyloxy)carbonyl)amino)-4-(*tert*-butoxy)-4-oxobutanamido)-*N*-methyl-3-(1-methyl-1*H*-indol-3-yl)propanamido)-5-(benzyloxy)-4-methylpentanamido)-6-((*tert*-butoxycarbonyl)(methyl)amino)hexan-amido)-3-methoxy-3-phenylpropanoyl)-L-valyl)-*N*-methyl-L-leucinate (8)

^{1}H-NMR (500 MHz, DMSO-d$_6$, 373 K):

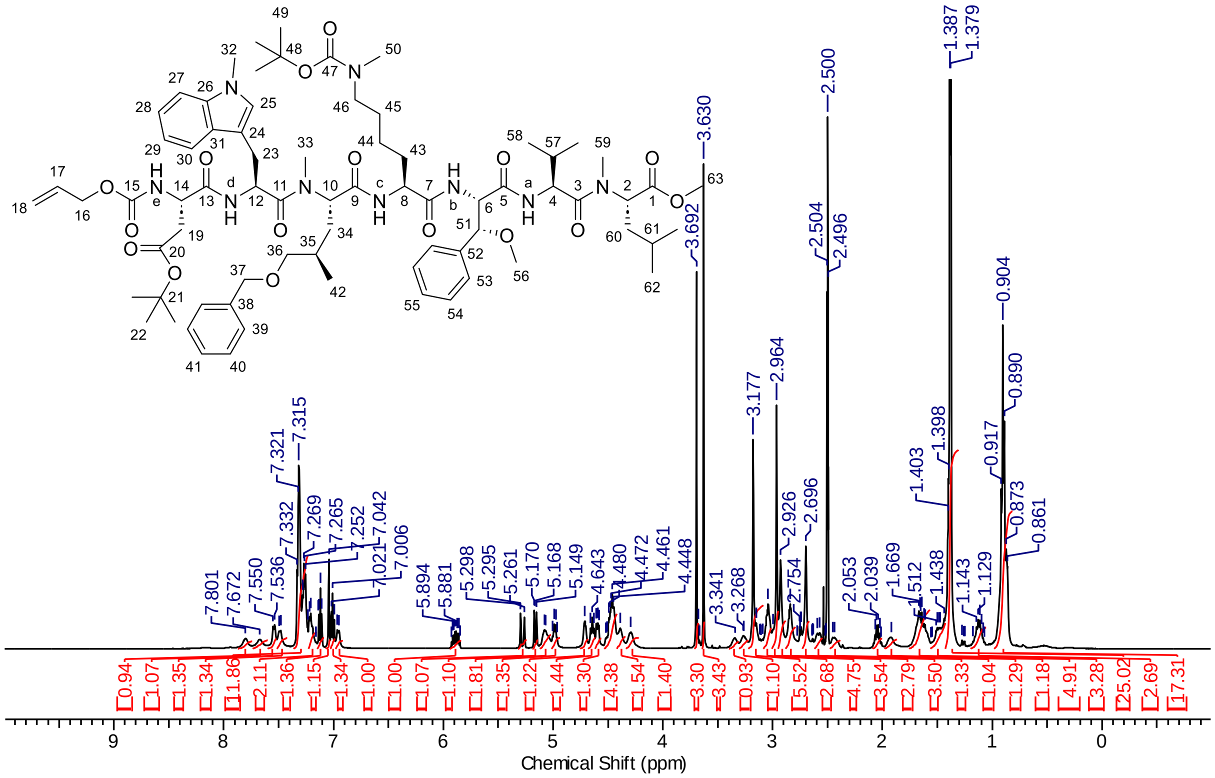

^{13}C-NMR (125 MHz, DMSO-d$_6$, 373 K):

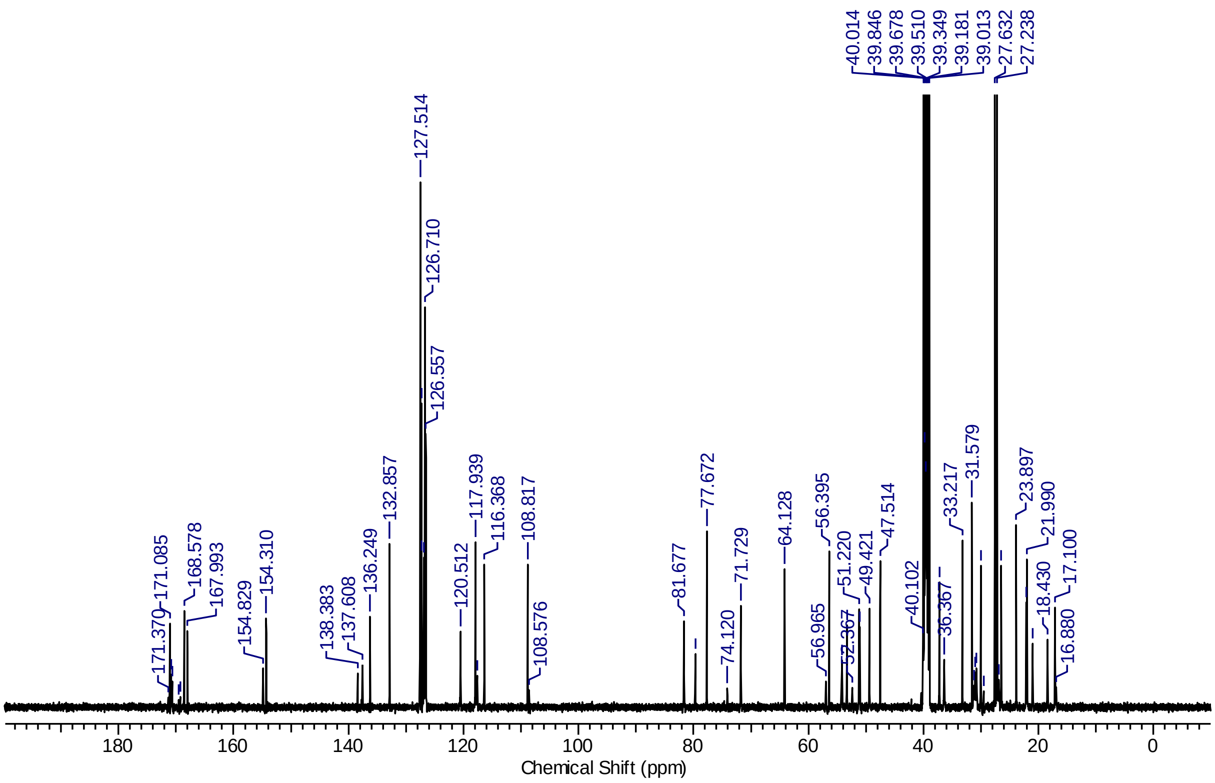

tert-Butyl 2-((2*S*,5*S*,8*S*,11*S*,14*S*,17*S*,20*S*)-8-((*R*)-3-(benzyloxy)-2-methylpropyl)-11-(4-((_tert_-butoxycarbonyl)(methyl)amino)butyl)-20-isobutyl-17-isopropyl-14-((*R*)-methoxy(phenyl)methyl)-7,19-dimethyl-5-((1-methyl-1*H*-indol-3-yl)methyl)-3,6,9,12,15,18,21-heptaoxo-1,4,7,10,13,16,19-heptaazacyclohenicosan-2-yl)acetate (9)

^{1}H-NMR (400 MHz, DMSO-d$_6$):

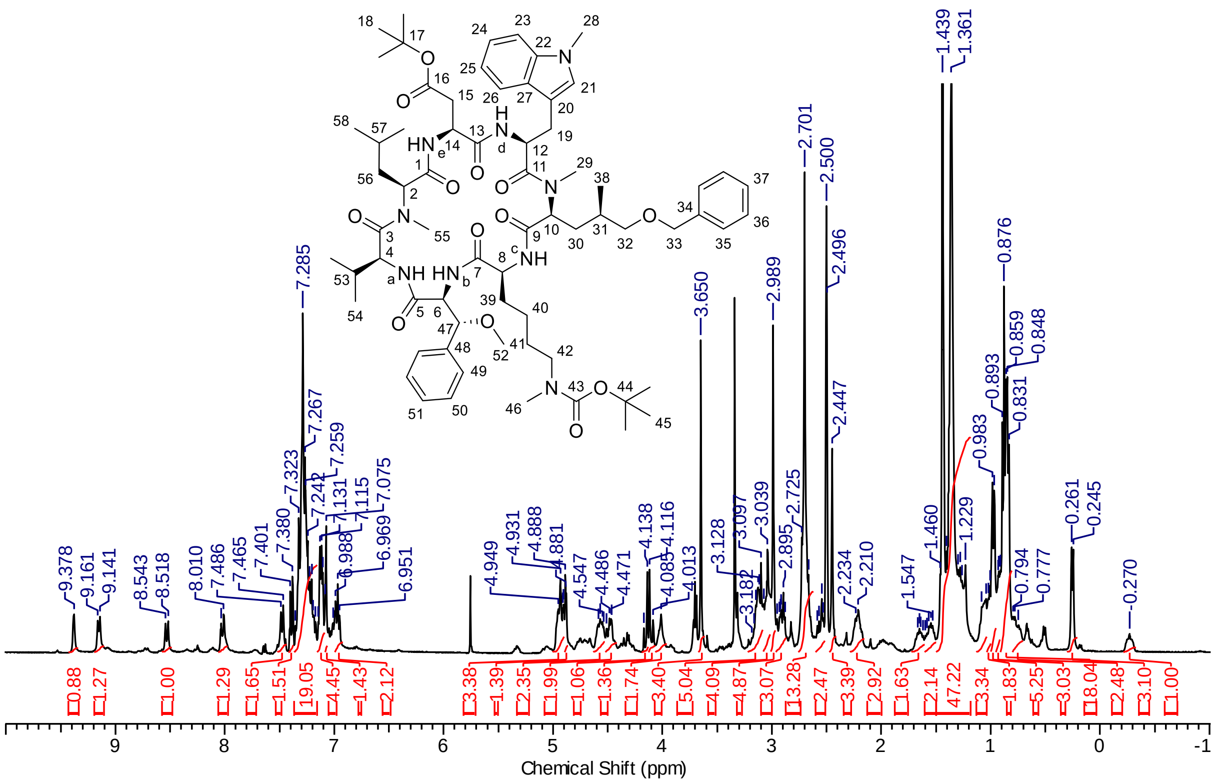

^{13}C-NMR (100 MHz, DMSO-d$_6$):

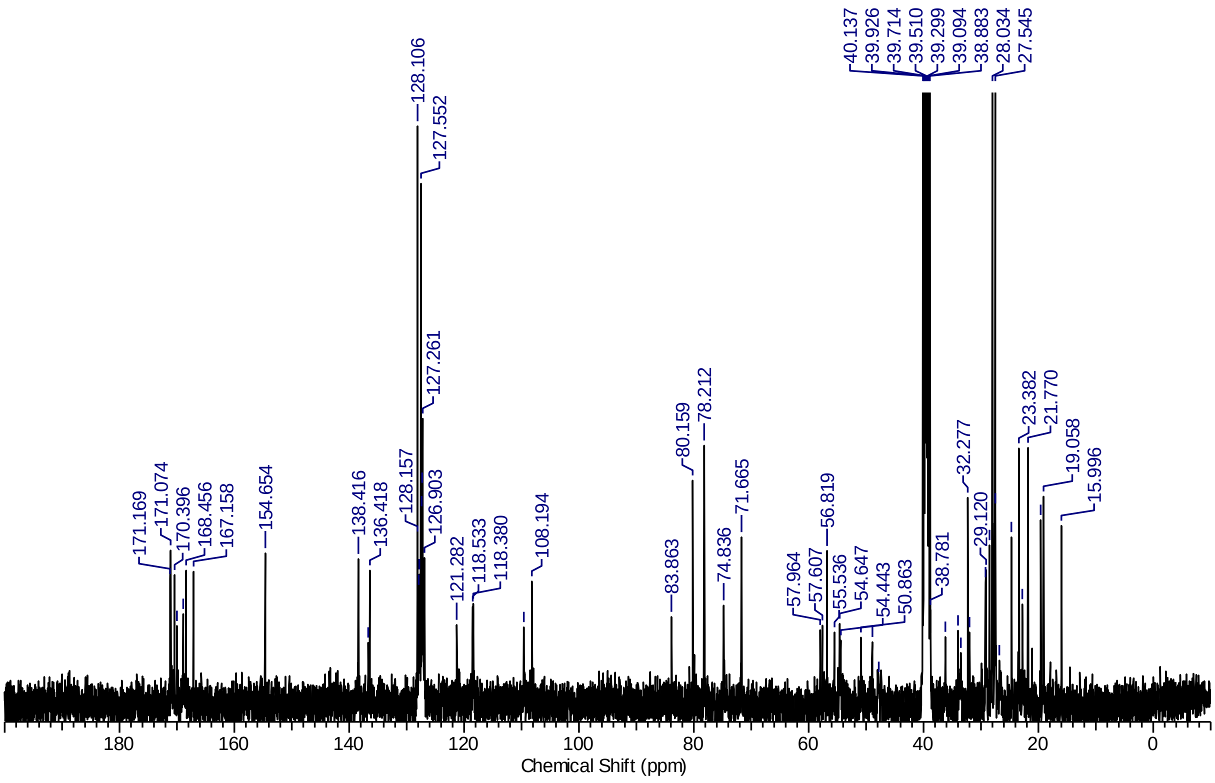

(1*S*,4*S*,7*S*,10*S*,13*S*,16*S*,19*S*)-16-((*R*)-3-(Benzyloxy)-2-methylpropyl)-4-isobutyl-7-isopropyl-10-((*R*)-methoxy(phenyl)methyl)-5,17,24-trimethyl-19-((1-methyl-1*H*-indol-3-yl)methyl)-2,5,8,11,14,17,20,24-octaazabicyclo[11.8.7]octacosan-3,6,9,12,15,18,21,23-octaone (10)

^{1}**H-NMR** (500 MHz, DMSO-d_6):

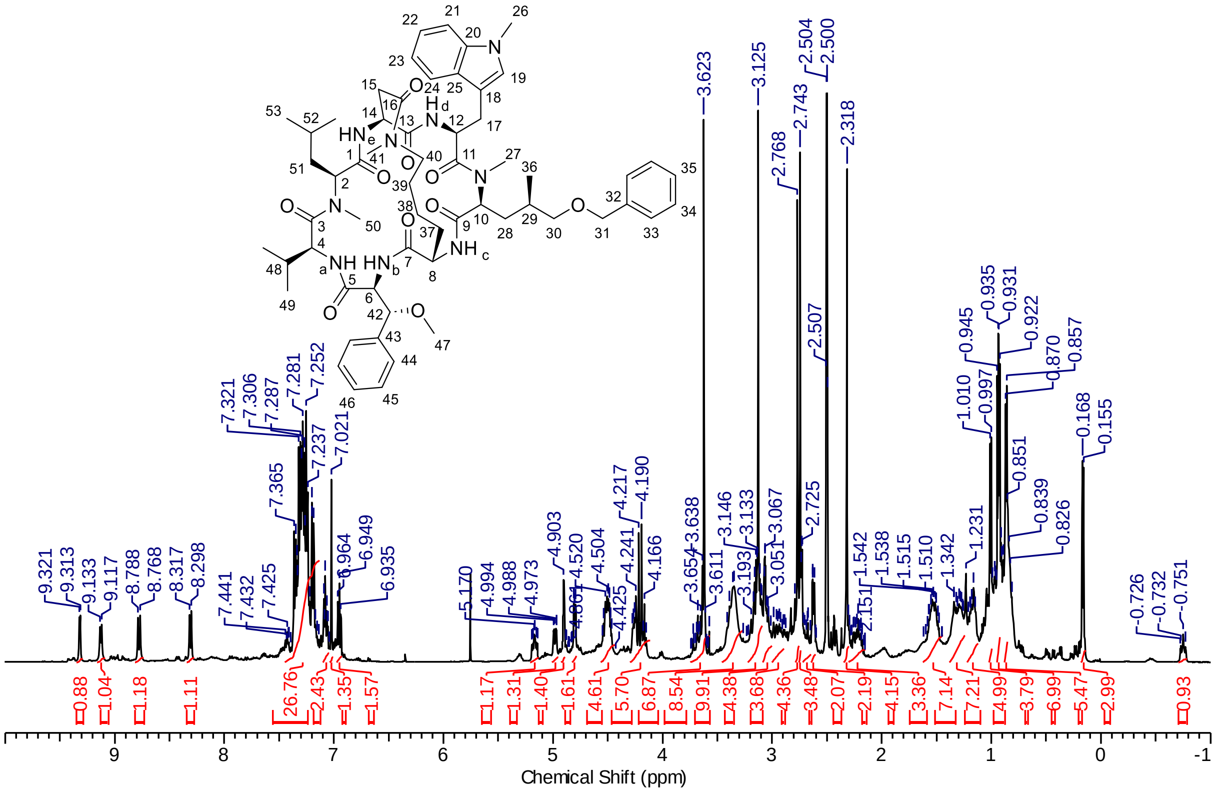

^{13}**C-NMR** (125 MHz, DMSO-d_6):

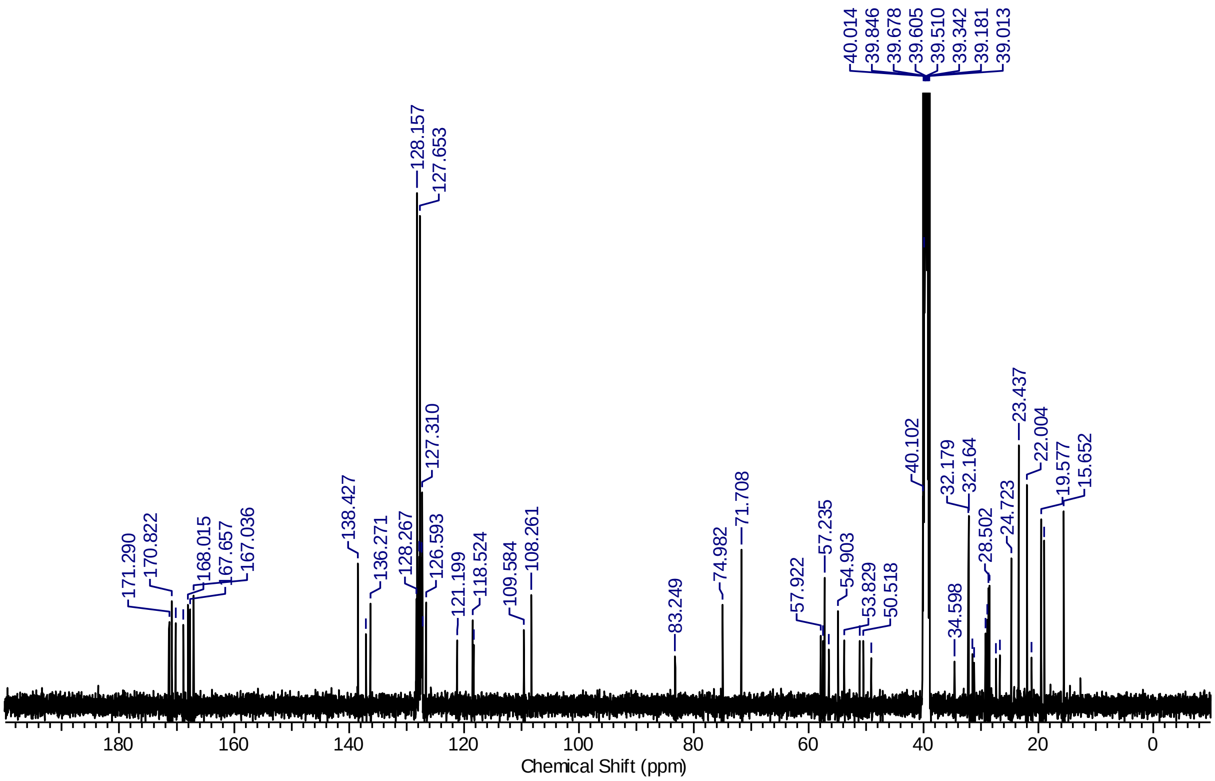

(1*S*,4*S*,7*S*,10*S*,13*S*,16*S*,19*S*)-16-((*R*)-3-Hydroxy-2-methylpropyl)-4-isobutyl-7-isopropyl-10-((*R*)-methoxy(phenyl)methyl)-5,17,24-trimethyl-19-((1-methyl-1*H*-indol-3-yl)methyl)-2,5,8,11,14,17,20,24-octaazabicyclo[11.8.7]octacosan-3,6,9,12,15,18,21,23-octaone (1)

^{1}H-NMR (500 MHz, $CDCl_3$):

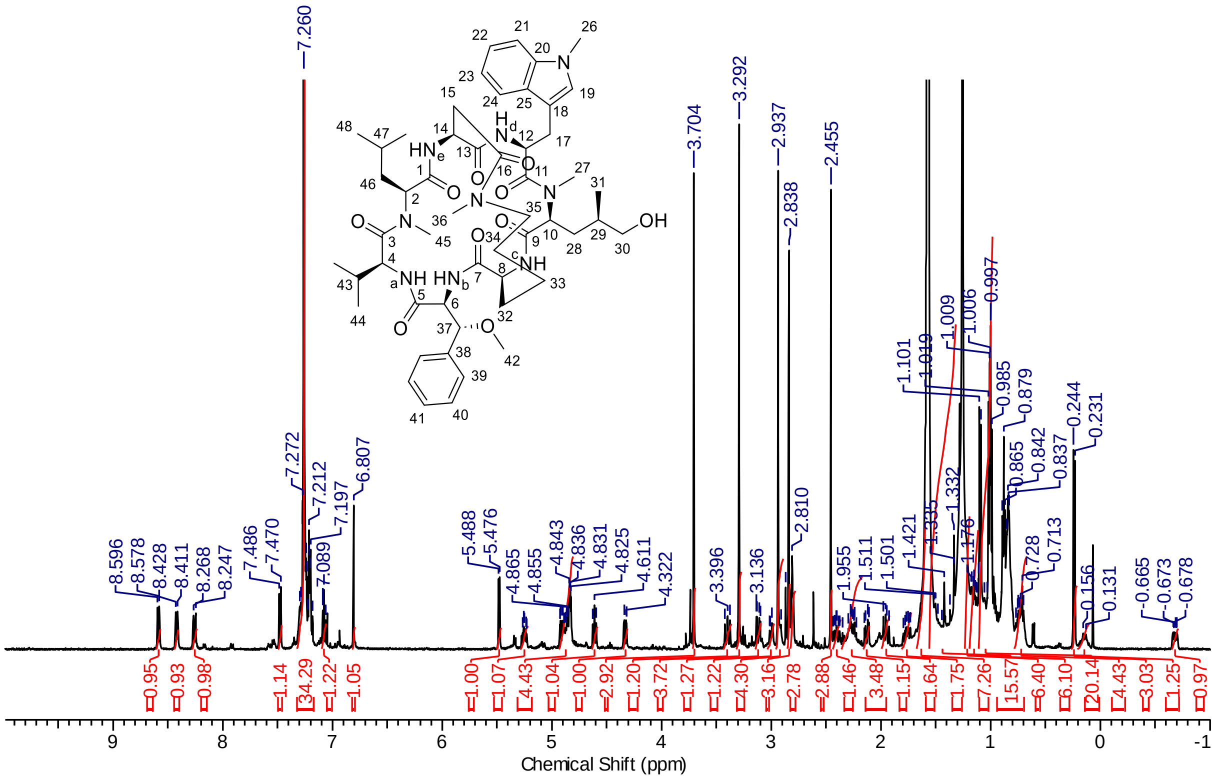

^{13}C-NMR (125 MHz, $CDCl_3$):

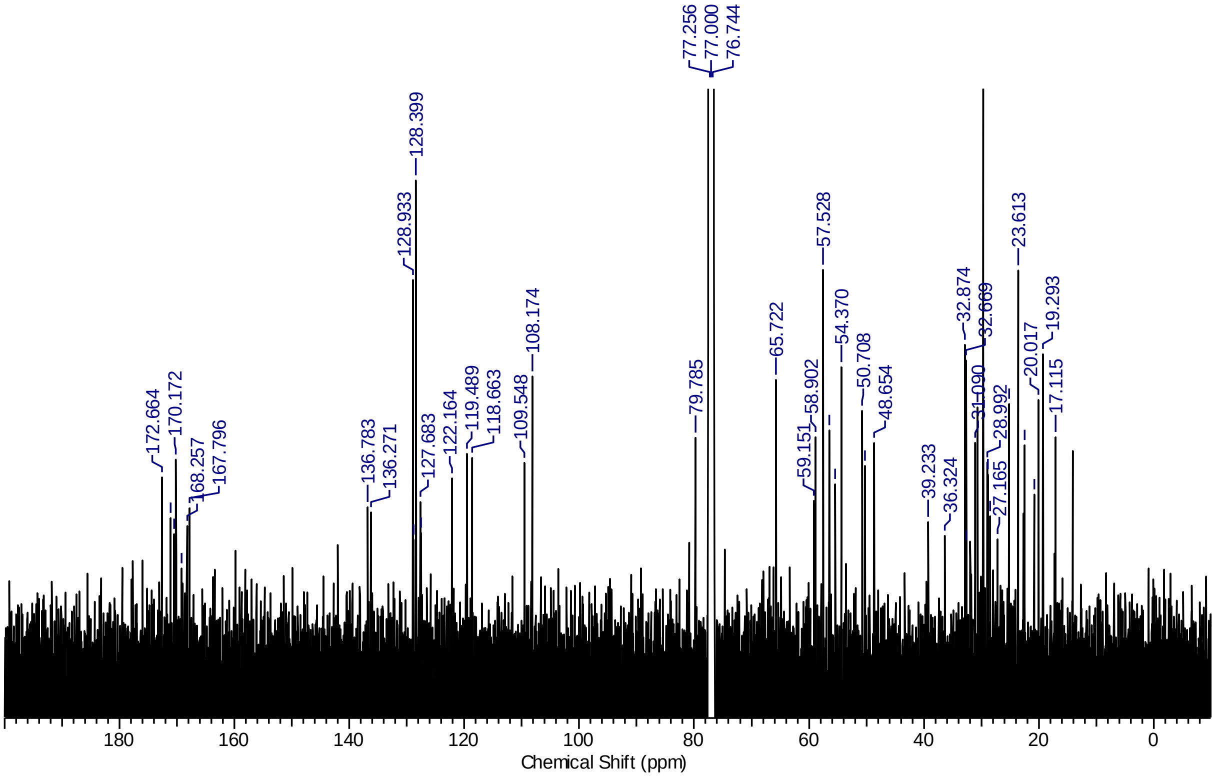

References

[1] a) C.-B. Xue, M. E. Voss, D. J. Nelson, J. J. W. Duan, R. J. Cherney, I. C. Jacobson, X. He, J. Roderick, L. Chen, R. L. Corbett, L. Wang, D. T. Meyer, K. Kennedy, W. F. DeGrado, K. D. Hardman, C. A. Teleha, B. D. Jaffee, R.-Q. Liu, R. A. Copeland, M. B. Covington, D. D. Christ, J. M. Trzaskos, R. C. Newton, R. L. Magolda, R. R. Wexler, C. P. Decicco, *J. Med. Chem.* **2001**, *44*, 2636–2660; b) D. C. Tully, A. K. Chatterjee, A. Vidal, H. M. J. Petrassi, Z. Wang, B. Bursulaya, G. Spraggon, *US 2007/0275906 A1*, **2007**.

[2] C. A. A. Van Boeckel, R. C. Buijsman, M. De Kort, D. G. Meuleman, *EP 1574516 A1*, **2005**.

[3] S. Chambon, S. Talano, C. Millois, L. Dumais, R. Pierre, L. Tomas, C. Mathieu, A.-L. Ghilini, N. Vanthuyne, K. Reverse, A. Brethon, V. Rodeschini, C. Comino, G. Mouis, G. El-Bazbouz, L. Clary, J.-F. Fournier, C. Bouix-Peter, C. S. Harris, L. F. Hennequin, *Tetrahedron* **2018**, *74*, 4805–4822.

[4] P. Barbie, U. Kazmaier, *Org. Lett.* **2016**, *18*, 204–207.

[5] A. Kiefer, U. Kazmaier, *Org. Biomol. Chem.* **2019**, *17*, 88–102.

[6] L. Junk, U. Kazmaier, *Angew. Chem. Int. Ed.* **2018**, *57*, 11432–11435.

[7] K. C. Nicolaou, A. A. Estrada, M. Zak, S. H. Lee, B. S. Safina, *Angew. Chem. Int. Ed.* **2005**, *44*, 1378–1382.

[8] S. Lemaire-Audoire, M. Savignac, E. Blart, G. Pourcelot, J. P. Genêt, J.-M. Bernard, *Tetrahedron Lett.* **1994**, *35*, 8783–8786.

[9] J. S. Panek, F. Xu, *J. Am. Chem. Soc.* **1995**, *117*, 10587–10588.
